# A substantial prehistoric European ancestry amongst Ashkenazi maternal lineages

**DOI:** 10.1038/ncomms3543

**Published:** 2013-10-08

**Authors:** Marta D. Costa, Joana B. Pereira, Maria Pala, Verónica Fernandes, Anna Olivieri, Alessandro Achilli, Ugo A. Perego, Sergei Rychkov, Oksana Naumova, Jiři Hatina, Scott R. Woodward, Ken Khong Eng, Vincent Macaulay, Martin Carr, Pedro Soares, Luísa Pereira, Martin B. Richards

**Affiliations:** 1 Institute of Integrative and Comparative Biology, Faculty of Biological Sciences, University of Leeds, Leeds LS2 9JT, UK; 2 IPATIMUP (Instituto de Patologia e Imunologia Molecular da Universidade do Porto), Porto 4200-465, Portugal; 3 School of Applied Sciences, University of Huddersfield, Queensgate, Huddersfield HD1 3DH, UK; 4 Dipartimento di Biologia e Biotecnologie, Università di Pavia, Pavia 27100, Italy; 5 Dipartimento di Chimica, Biologia e Biotecnologie, Università di Perugia, Perugia 06123, Italy; 6 Sorenson Molecular Genealogy Foundation, Salt Lake City, Utah 84115, USA; 7 Vavilov Institute of General Genetics, Moscow 119991, Russia; 8 Charles University, Medical Faculty in Pilsen, Institute of Biology, CZ-301 66 Pilsen, Czech Republic; 9 Ancestry, Provo, Utah 84604, USA; 10 Centre for Global Archaeological Research, Universiti Sains Malaysia, 11800 USM Penang, Malaysia; 11 School of Mathematics and Statistics, University of Glasgow, Glasgow G12 8QQ, UK; 12 Faculdade de Medicina da Universidade do Porto, Porto 4200-319, Portugal; 13 These authors contributed equally to this work

## Abstract

The origins of Ashkenazi Jews remain highly controversial. Like Judaism, mitochondrial DNA is passed along the maternal line. Its variation in the Ashkenazim is highly distinctive, with four major and numerous minor founders. However, due to their rarity in the general population, these founders have been difficult to trace to a source. Here we show that all four major founders, ~40% of Ashkenazi mtDNA variation, have ancestry in prehistoric Europe, rather than the Near East or Caucasus. Furthermore, most of the remaining minor founders share a similar deep European ancestry. Thus the great majority of Ashkenazi maternal lineages were not brought from the Levant, as commonly supposed, nor recruited in the Caucasus, as sometimes suggested, but assimilated within Europe. These results point to a significant role for the conversion of women in the formation of Ashkenazi communities, and provide the foundation for a detailed reconstruction of Ashkenazi genealogical history.

## Abstract (web summary)

Ashkenazi mitochondrial DNA variation has four major founders whose sources are difficult to trace due to the rarity of Ashkenazi Jews in the general population. Here, the authors provide evidence that all four major founders originated from Europe and provide a genealogical record of the Ashkenazi.

## Graphical abstract

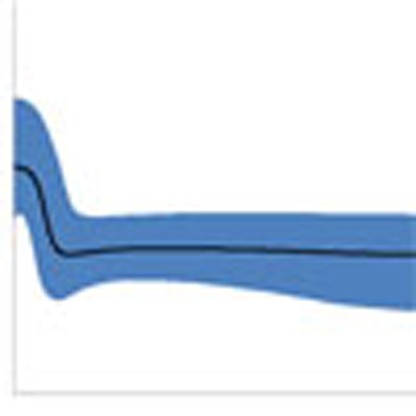

The origins of Ashkenazi Jews—the great majority of living Jews—remain highly contested and enigmatic to this day^1^,^2^,^3^,^4^,^5^,^6^,^7^,^8^,^9^,^10^,^11^. The Ashkenazim are Jews with a recent ancestry in central and Eastern Europe, in contrast to Sephardim (with an ancestry in Iberia, followed by exile after 1492), Mizrahim (who have always resided in the Near East) and North African Jews (comprising both Sephardim and Mizrahim). There is consensus that all Jewish Diaspora groups, including the Ashkenazim, trace their ancestry, at least in part, to the Levant, ~2,000–3,000 years ago^5^,^12^,^13^,^14^. There were Diaspora communities throughout Mediterranean Europe and the Near East for several centuries prior to the destruction of the Second Temple in Jerusalem in 70 CE (Common Era), and some scholars suggest that their scale implies proselytism and wide-scale conversion, although this view is very controversial^9^,^15^.

The Ashkenazim are thought to have emerged from dispersals north into the Rhineland of Mediterranean Jews in the early Middle Ages, although there is little evidence before the twelfth century^5^,^15^. After expulsions from Western Europe between the thirteenth and fifteenth centuries, the communities are thought to have expanded eastwards, especially in Poland, Lithuania and then Russia. The implied scale of this expansion has led some to argue, again very controversially, for mass conversions in the Khazar kingdom, in the North Caucasus region to the north and east of the Black Sea, following the Khazar leadership’s adoption of Judaism between the ninth and tenth centuries CE^8^,^9^.

We are then faced with several competing models for Ashkenazi origins: a Levantine ancestry; a Mediterranean/west European ancestry; a North Caucasian ancestry; or, of course, a blend of these. This seems an ideal problem to tackle with genetic analysis, but after decades of intensive study a definitive answer remains elusive. Although we might imagine that such an apparently straightforward admixture question might be readily addressed using genome-wide autosomal markers, recent studies have proposed contradictory conclusions. Several suggest a primarily Levantine ancestry with south/west European admixture^3^,^4^, but another concludes that the ancestry is largely Caucasian^16^, implying a major source from converts in the Khazar kingdom^17^. An important reason for disagreement is that the Ashkenazim have undergone severe founder effects during their history, drastically altering the frequencies of genetic markers and distorting the relationship with their ancestral populations.

This problem can be resolved by reconstructing the relationships genealogically, rather than relying on allele frequencies, using the non-recombining marker systems: the paternally inherited male-specific part of the Y chromosome (MSY) and the maternally inherited mitochondrial DNA (mtDNA). This kind of analysis can be very powerful, because nesting of particular lineages within clusters from a particular geographical region allows us to pinpoint the source for those lineages, by applying the parsimony principle. This has indeed been attempted, with the MSY results interpreted plausibly to suggest an overwhelming majority of Near Eastern ancestry on the Ashkenazi male line of descent^11^,^18^,^19^,^20^,^21^, albeit with much higher levels (>50%) of European (potentially east European) lineages in Ashkenazi Levites^22^, suggesting a possible Khazar source in that particular case.

The maternal line has also been studied, and indeed Ashkenazi mtDNAs are highly distinctive, but they have proved difficult to assign to a source population^1^,^2^,^11^. Some progress has been made by targeting whole-mtDNA genomes or mitogenomes, which provide much higher genealogical (and therefore geographical) and chronological resolution than the control-region sequences used previously—although the far larger control-region database remains an invaluable guide to their geographic distribution. Using this approach, Behar *et al.*^2^ identified four major founder clusters, three within haplogroup K—amounting to 32% of sampled Ashkenazi lineages—and one within haplogroup N1b, amounting to another 9%. These lineages are extremely infrequent across the Near East and Europe, making the identification of potential source populations very challenging. Nevertheless, they concluded that all four most likely arose in the Near East and were markers of a migration to Europe of people ancestral to the Ashkenazim only ~2,000 years ago^1^,^2^. The remaining ~60% of mtDNA lineages in the Ashkenazim remained unassigned to any source, with the exception of the minor haplogroup U5 and V lineages (~6% in total), which implied European ancestry^1^,^23^.

Here we focus on both major and minor founders, with a much larger database from potential source populations. We first analyse 956 (72 newly generated) mitogenomes from haplogroup U8 (including 909 from haplogroup K, U8’s major subclade): 477 of these are from Europe and 106 from the Near East/Caucasus. We show that European and Near Eastern lineages largely fall into discrete, ancient clusters, with minor episodes of gene flow, suggesting that haplogroup K diversified separately in Europe and the Near East during the last glacial period. Of the three Ashkenazi founders, K1a1b1a and K1a9 were most likely assimilated in west (perhaps Mediterranean) Europe and K2a2a1 in west/central Europe. Most surprisingly, by analysing two new N1b2 sequences selected from a database of 278 N1b HVS-I sequences, in the context of 44 published N1b sequences^24^, we show that the highly distinctive N1b2 subclade, making up another 9% of Ashkenazi lineages, was likely assimilated in Mediterranean Europe, rather than in the Near East as previously proposed^2^. Moreover, from a survey of another >2,500 complete mtDNA genomes and >28,000 control-region sequences from Europe, the Near East and the Caucasus, in comparison with the available database of 836 Ashkenazi control-region sequences and a handful of published mitogenomes, we also evaluate the minor founders. Overall, we estimate that most (>80%) Ashkenazi mtDNAs were assimilated within Europe. Few derive from a Near Eastern source, and despite the recent revival of the ‘Khazar hypothesis’^16^, virtually none are likely to have ancestry in the North Caucasus. Therefore, whereas on the male side there may have been a significant Near Eastern (and possibly east European/Caucasian) component in Ashkenazi ancestry, the maternal lineages mainly trace back to prehistoric Western Europe. These results emphasize the importance of recruitment of local women and conversion in the formation of Ashkenazi communities, and represent a significant step in the detailed reconstruction of Ashkenazi genealogical history.

## Results

### Four major founder lineages within haplogroup K and N1b

**Figure 1:**
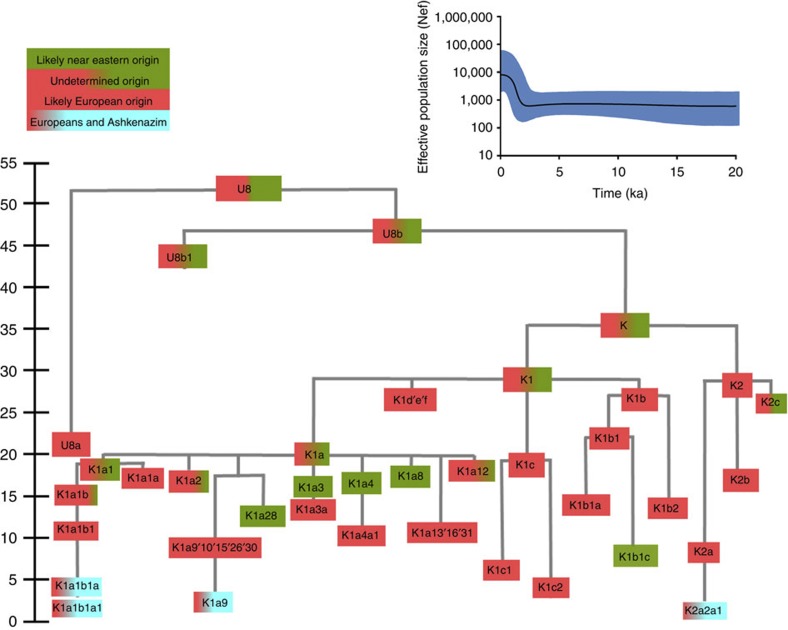
Inferred ancestry of the main subclades within haplogroup U8. The timescale (ka) is based on ML estimations for mitogenomes. Inset: Bayesian skyline plot of 34 Ashkenazi haplogroup K lineages, showing growth in effective population size (*N*_*ef*_) over time.

**Figure 2:**
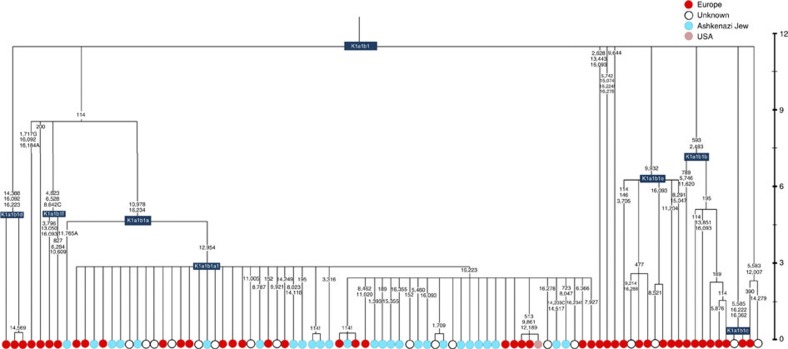
Phylogenetic tree of haplogroup K1a1b1. Time scale (ka) based on ML estimations for mitogenome sequences.

Haplogroup K arose within haplogroup U8~36 ka, in Europe or the Near East, with the minor subclades K1b, K1c and K2 all most likely arising in Europe, between the last glacial period and the Neolithic (Fig. 1; Supplementary Note 1; Supplementary Data 1–3; Supplementary Figs S1–S3; Supplementary Tables S1–S3). K1a expanded from ~20 ka onwards, both in the Near East and Europe, with its major subclade, K1a1b1 (Fig. 2), mainly restricted to Europe (with a few instances in North Africa), arriving from the Near East by ~11.5 ka, the beginning of the Holocene (Supplementary Note 1).

**Figure 3:**
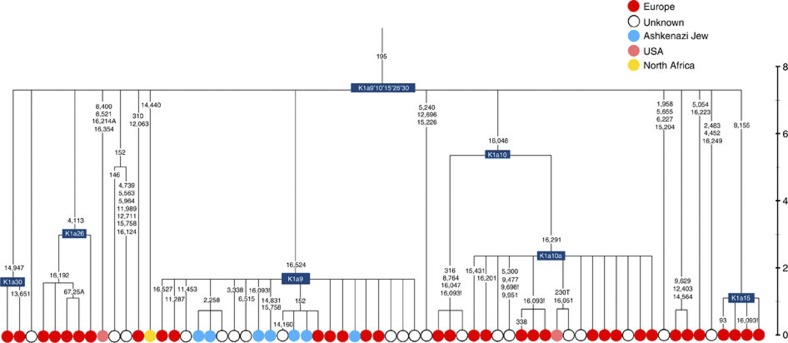
Phylogenetic tree of haplogroup K1a9 in the context of the putative clade K1a9′10′15′26′30. Time scale (ka) based on ML estimations for mitogenome sequences.

**Figure 4:**
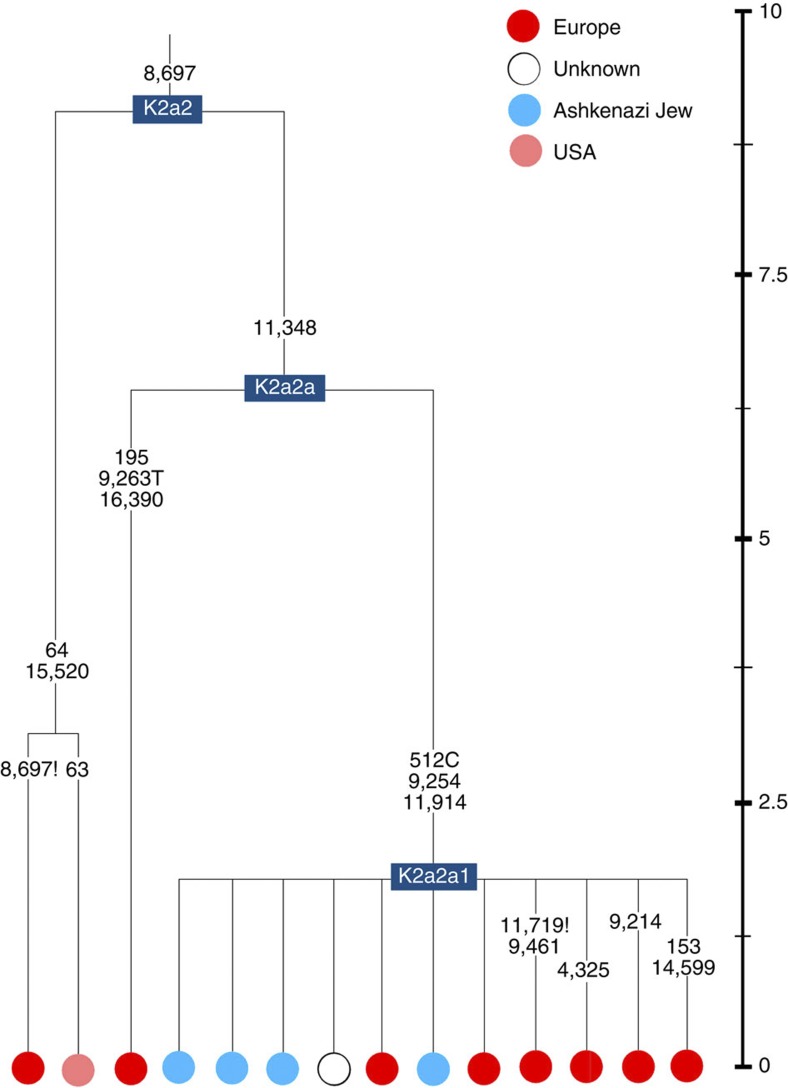
Phylogenetic tree of haplogroup K2a2. Time scale (ka) based on ML estimations for mitogenome sequences.

Almost half of mtDNAs in west/central European Ashkenazi Jews belong to haplogroup K, declining to ~15% in east European Jews^1^,^11^, with almost all falling into three subclades: K1a1b1a, K1a9 and K2a2a1^2^,^25^ (Figs 1, 2, 3, 4; Supplementary Fig. S4). These three founder clusters show a strong expansion signal beginning ~2.3 ka, with the overall effective population size for these lineages increasing 13-fold by 275 years ago (Fig.1).

**Figure 5:**
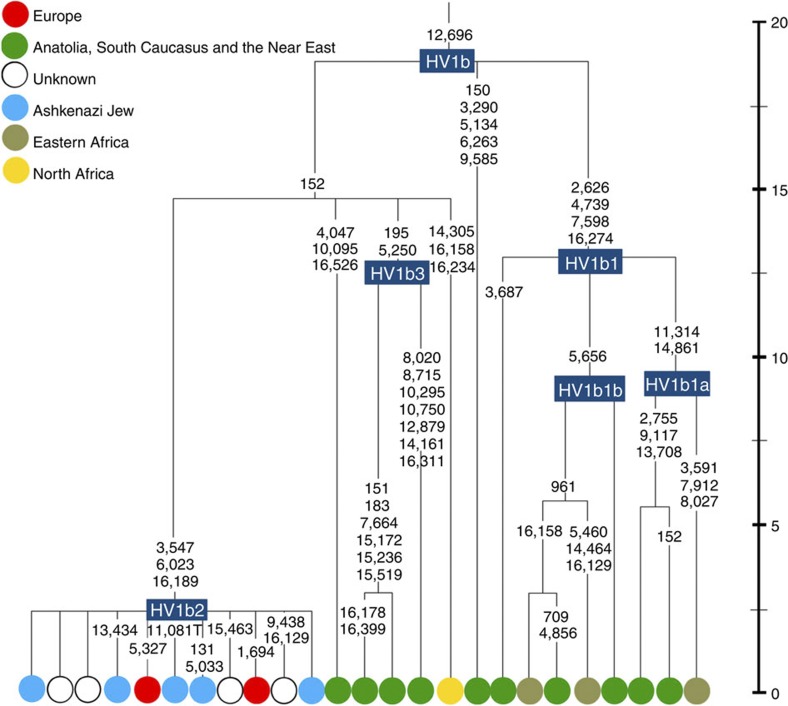
Phylogenetic tree of haplogroup HV1b. Time scale (ka) based on ML estimations for mitogenome sequences.

K1a1b1a (slightly re-defined, due to the improved resolution of the new tree) (Fig. 2) accounts for 63% of Ashkenazi K lineages (or ~20% of total Ashkenazi lineages) and dates to ~4.4 ka with maximum likelihood (ML); however, all of the samples within it, except for one, nest within a further subclade, K1a1b1a1, dating to ~2.3 ka (Supplementary Data 2). K1a1b1a1 is also present in non-Ashkenazi samples, mostly from central/east Europe. As they are nested by Ashkenazi lineages, these are likely due to gene flow from Ashkenazi communities into the wider population. The pattern of gene flow out into the neighbouring communities is seen in the other two major K founders, and also in haplogroups H and J; it is especially clear when the nesting and nested populations are more distinct, for example in the case of haplogroup HV1b, which has a deep ancestry in the Near East (Fig. 5; Supplementary Table S4).

The K1a1b1 lineages within which the K1a1b1a sequences nest (including 19 lineages of known ancestry) are solely European, pointing to an ancient European ancestry. The closest nesting lineages are from Italy, Germany and the British Isles, with other subclades of K1a1b1 including lineages from west and Mediterranean Europe and one Hutterite (Hutterites trace their ancestry to sixteenth-century Tyrol)^26^. Typing/HVS-I results have also indicated several from Northwest Africa, matching European HVS-I types^2^, likely the result of gene flow from Mediterranean Europe. K1a1b1a is also present at low frequencies in Spanish-exile Sephardic Jews, but absent from non-European Jews, including a database of 289 North African Jews^2^,^25^. Notably, it is not seen in Libyan Jews^25^, who are known to have a distinct Near Eastern ancestry, with no known influx from Spanish-exile immigrants (although Djerban Jews, with a similar history, have not been tested to date for mtDNA, they closely resemble Libyan Jews in autosomal analyses^27^). Thus the Ashkenazi subclade of K1a1b1 most likely had a west European source.

K1a9 (Fig. 3; Supplementary Fig. S4), accounting for another 20% of Ashkenazi K lineages (or 6% of total Ashkenazi lineages) and also dating to ~2.3 ka with ML (Supplementary Data 2) again includes both Ashkenazi and non-Ashkenazi lineages solely from east Europeans (again suggesting gene flow out into the wider communities). Like K1a1b1a, it is also found, at much lower frequencies, in Sephardim. Here the ancestral branching relationships are less clear (Supplementary Note 1 and Supplementary Fig. S4), but K1a9 is most plausibly nested within the putative clade K1a9′10′15′26′30, dating to ~9.8 ka, which otherwise includes solely west European (and one Tunisian) lineages, again pointing to a west European source.

K2a2 (Fig. 4) accounts for another 16% of Ashkenazi K lineages (or ~5% of total Ashkenazi lineages) and dates to ~8.4 ka (Supplementary Data 2). Ashkenazi lineages are once more found in a shallow subclade, K2a2a1, dating to ~1.5 ka, that otherwise again includes only east Europeans, suggesting gene flow from the Ashkenazim. Conversely, the nesting clades, K2a2 and K2a2a, although poorly sampled, include only French and German lineages. K2a2a is not found in non-European Jews^25^.

Haplogroup K is rarer in the North Caucasus than in Europe or the Near East (<4% (ref. 23)) and the three Ashkenazi founder clades have not been found there (Supplementary Note 2). We tested all eight K lineages out of 208 samples from the North Caucasus, and all belonged to the Near Eastern subclades K1a3, K1a4 and K1a12. Haplogroup K is more common in Chuvashia, but those sampled belong to K1a4, K1a5 and pre-K2a8.

The fourth major Ashkenazi founder mtDNA falls within haplogroup N1b (ref. 2). The distribution of N1b is much more focused on the Near East than that of haplogroup K (ref. 24), and the distinctive Ashkenazi N1b2 subclade has accordingly being assigned to a Levantine source^2^. N1b2 has until now been found exclusively in Ashkenazim, and although it dates to only ~2.3 ka, it diverged from other N1b lineages ~20 ka (ref. 24) (Supplementary Table S5). N1b2 can be recognized in the HVS-I database by the variant 16176A, but Behar *et al.*^2^ tested 14 Near Eastern samples (and some east Europeans) with this motif and identified it as a parallel mutation. Therefore, despite the long branch leading to N1b2, no Near Eastern samples are known to belong to it.

**Figure 6:**
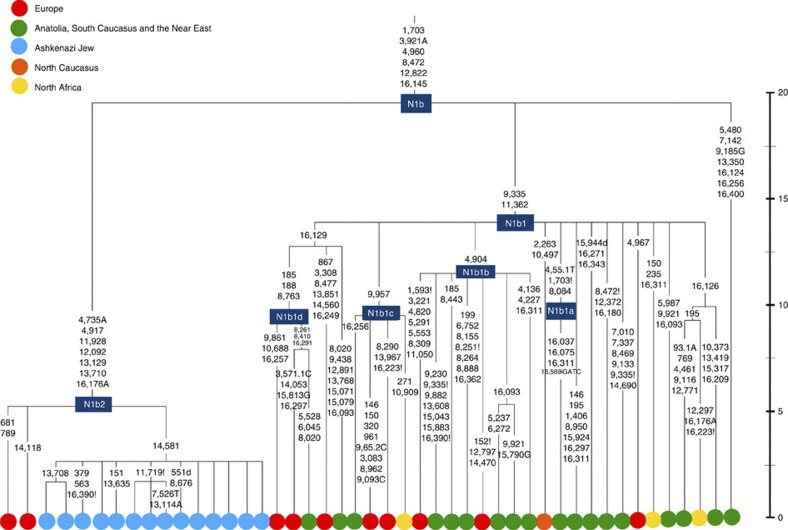
Phylogenetic tree of haplogroup N1b. Time scale (ka) based on ML estimations for mitogenome sequences.

In our unpublished database of 6991 HVS-I sequences, however, we identified two Italian samples with the 16176A marker, which we completely sequenced. We confirmed that they belong to N1b2 but diverge before the Ashkenazi lineages ~5 ka, nesting the Ashkenazi cluster (Fig. 6; Supplementary Table S5). This striking result suggests that the Italian lineages may be relicts of a dispersal from the Near East into Europe before 5 ka, and that N1b2 was assimilated into the ancestral Ashkenazi population on the north Mediterranean ~2 ka. Although we found only two samples suggesting an Italian ancestry for N1b2, the control-region database available for inspection is very large (28,418 HVS-I sequences from Europe, the Near East and the Caucasus, of which 278, or ~1%, were N1b). Moreover, the conclusion is supported by our previous founder analysis of N1b HVS-I sequences, which dated the dispersal into Europe to the late Pleistocene/early Holocene^24^.

### Minor Ashkenazi mtDNA lineages

There is now a large number of mitogenomes from Europe, the Caucasus and the Near East (~3,500, with >70 Ashkenazim), and a substantial Ashkenazi mtDNA control-region database of 836 samples^1^,^2^,^11^ (Supplementary Table S6). We therefore endeavoured to cross-reference the two in order to pinpoint most of the control-region data within the mitogenome phylogeny.

**Figure 7:**
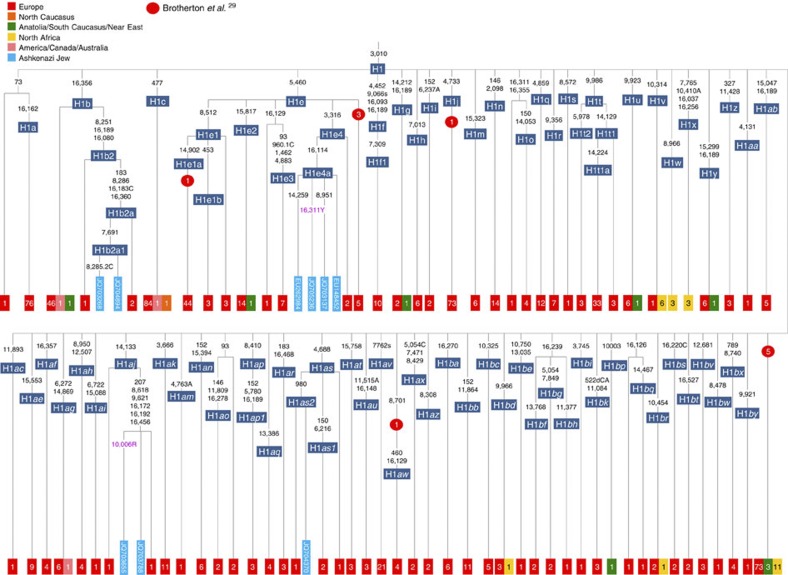
Schematic phylogenetic tree of haplogroup H1. Only the Ashkenazi lineages are shown in full detail; the distribution of other lineages is indicated using small squares by the number present in the full tree for each subclade. Prehistoric European (all Neolithic, except for the H1aw lineage, which dates to the Iron Age) lineages are shown using red circles^29^.

**Figure 8:**
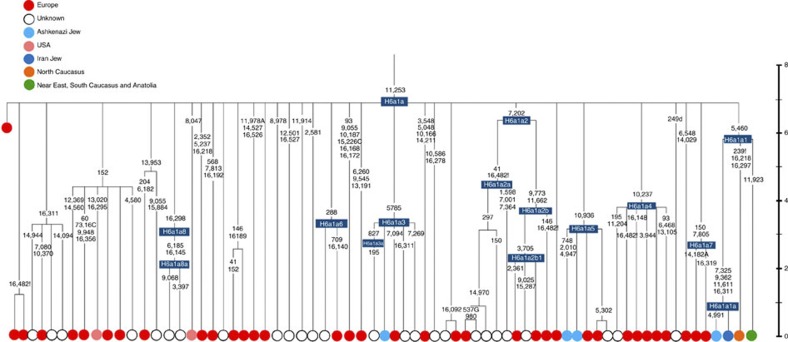
Phylogenetic tree of Ashkenazi founders within haplogroup H6a1a. Time scale (ka) based on ML estimations for mitogenome sequences. A Late Neolithic Corded Ware lineage from central Europe^29^ is shown in red emerging directly from the root.

Besides the four haplogroup K and N1b founders, the major haplogroup in Ashkenazi Jews is haplogroup H, at 23% of Ashkenazi lineages, which is also the major haplogroup in Europeans (40–50% in Europe, ~25% in the North Caucasus and ~19% in the Near East)^28^. There are 29 Ashkenazi H mitogenomes available (Supplementary Table S7), 26 (90%) of which nest comfortably within European subclades dating to the early Holocene (Supplementary Note 3, Figs 7 and 8; Supplementary Figs S5–S10; Supplementary Table S8). Most, in fact, nest more specifically within west/central European subclades, with closely matching sequences in east Europe, as with the pattern for the K founder clades. The Ashkenazi mitogenomes from haplogroup H include 39% belonging to H1 or H3, which are most frequent in west Europe and rare outside Europe. The nesting relationships in some cases point (albeit tentatively) to a central European source, but in many cases comparison with the HVS-I database indicates matches in west Europe. The phylogeographic conclusions based on the nesting relationships are strongly supported for haplogroup H by evidence from the study of prehistoric remains, showing in almost all cases that the lineages concerned were present in Europe since at least the early Bronze Age, ~3.5 ka (Supplementary Table S7)^29^. There is no suggestion of assimilation from the North Caucasus, where most H lineages differ from those of Europe^23^ (Supplementary Note 2).

**Figure 9:**
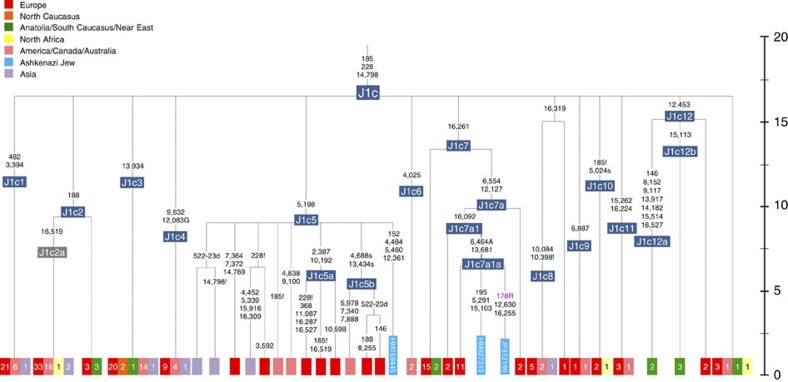
Schematic phylogenetic tree of haplogroup J1c. Only the Ashkenazi lineages are shown in full detail; the distribution of other lineages is indicated using small squares for each subclade with the number present in the full tree given in each case. For the full tree see Pala *et al.*^30^ Time scale (ka) based on ML estimations for mitogenome sequences.

Haplogroup J comprises 7% of the Ashkenazi control-region database. Around 72% of these can be assigned to J1c, now thought to have arisen within Late Glacial Europe^30^, and 19% belong to J1b1a1, also restricted to Europe. Thus >90% of the Ashkenazi J lineages have a European origin, with ~7% (J1b and J2b) less clearly associated. Many have a probable west/central European source, despite (like H) being most frequent in eastern Ashkenazim. The four Ashkenazi J mitogenomes, in J1c5, J1c7a1a and J1c7d, once again show a striking pattern of Mediterranean, west and central European lineages enclosing Ashkenazi/east European ones (Fig. 9).

Haplogroups U5, U4 and HV0 (6.3% between them overall) arose within Europe. Some of these lineages, which are again more frequent in the eastern than western Ashkenazi, may have been assimilated in central Europe. The haplogroup T lineages (5% overall) are more difficult to assign, but at least 60% (in T2a1b, T2b, T2e1 and T2e4) are likely of European and ~10% (T1b3 and T2a2) Near Eastern origin^30^. The haplogroup I lineages have evidently been present in Europe at least since the Neolithic, as indicated by both phylogeographic and ancient DNA analyses^31^. Haplogroup W3 may have originated in the Near East but spread to Europe as early as the Late Glacial^31^. The M1a1b lineage is characteristic of the north Mediterranean and was most likely assimilated there^32^, but the U6a and L2a1l lineages are more difficult to pin down.

The main lineages with a potentially Near Eastern source include HV1, R0a1a and U7a5 (~8.3% in all). HV1b2 mitogenomes, in particular, date to ~2 ka and nest within a cluster of Near Eastern HV1b lineages dating to ~18 ka (Fig. 5; Supplementary Table S4). Others such as U1a and U1b have an ultimately Near Eastern origin but, like N1b, have been subsequently distributed around the north Mediterranean. In general, it is more difficult to assign lineages to a Near Eastern source with confidence, as the much larger control-region database indicates that (as with N1b2) many lineages with deep Near Eastern ancestry became widely dispersed along the north Mediterranean during the Holocene, and may alternatively have been assimilated there.

**Figure 10:**
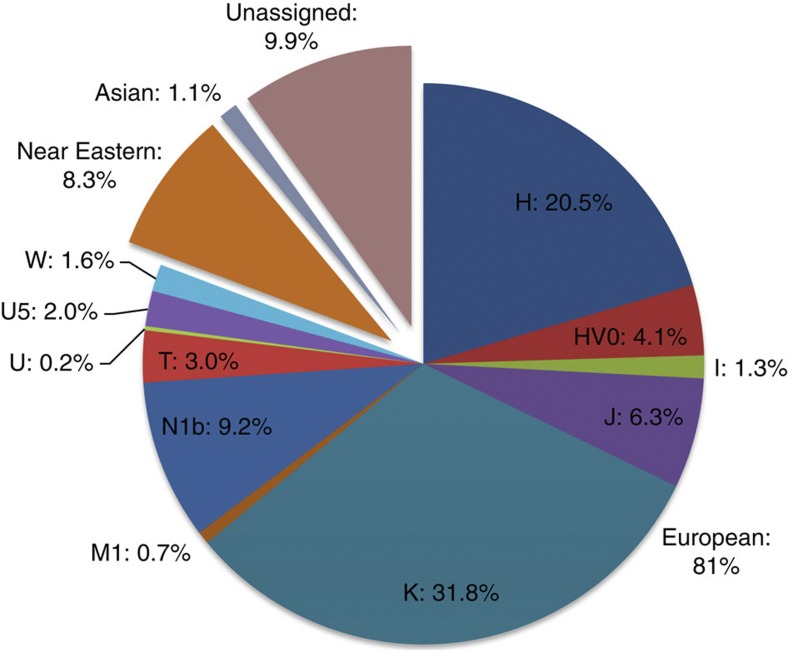
Estimated contributions of European mtDNA lineages to the Ashkenazi mtDNA pool shown by major haplogroup. The possible overall Near Eastern contribution and fraction of unassigned lineages are also indicated.

If we allow for the possibility that K1a9 and N1b2 might have a Near Eastern source, then we can estimate the overall fraction of European maternal ancestry at ~65%. Given the strength of the case for even these founders having a European source, however, our best estimate is to assign ~81% of Ashkenazi lineages to a European source, ~8% to the Near East and ~1% further to the east in Asia, with ~10% remaining ambiguous (Fig. 10; Supplementary Table S9). Thus at least two-thirds and most likely more than four-fifths of Ashkenazi maternal lineages have a European ancestry.

## Discussion

The extent to which Ashkenazi Jewry trace their ancestry to the Levant or to Europe is a long-standing question^5^, which remains highly controversial^3^,^4^,^6^,^12^,^13^,^14^,^16^,^17^. Our results, primarily from the detailed analysis of the four major haplogroup K and N1b founders, but corroborated with the remaining Ashkenazi mtDNAs, suggest that most Ashkenazi maternal lineages trace their ancestry to prehistoric Europe.

Previous researchers proposed a Levantine origin for the three Ashkenazi K founders from several indirect lines of evidence: shared ancestry with non-Ashkenazi Jews, shared recent ancestry with Mediterranean samples, and their absence from amongst non-Jews^2^, and this suggestion has been widely accepted^4^. However, our much more detailed analyses show that two of the major Ashkenazi haplogroup K lineages, K1a1b1a and K2a2a1 have a deep European ancestry, tracing back at least as far as the early and mid-Holocene respectively. They both belong to ancient European clades (K1a1b1 and K2) that include primarily European mtDNAs, to the virtual exclusion of any from the Near East. Despite some uncertainty in its ancestral branching relationships, a European ancestry seems likely for the third founder clade, K1a9. The heavy concentration of Near Eastern haplogroup K lineages within particular, distinct subclades of the tree, and indeed the lack of haplogroup K lineages in Samaritans, who might be expected to have shared an ancestral gene pool with ancient Israelites, both strongly imply that we are unlikely to have missed a hitherto undetected Levantine ‘reservoir’ of haplogroup K variation (Supplementary Note 1).

Furthermore, our results suggest that N1b2, for which a Near Eastern ancestry was proposed (with much greater confidence than for K) by Behar *et al.*^2^, is more likely to have been assimilated into the ancestors of the Ashkenazi in the north Mediterranean. Finally, our cross-comparison of control-region and mitogenome databases shows that the great majority of the remaining ~60% of Ashkenazi lineages, belonging to haplogroups H, J, T, HV0, U4/U5, I, W and M1 also have a predominantly European ancestry.

Overall, it seems that at least 80% of Ashkenazi maternal ancestry is due to the assimilation of mtDNAs indigenous to Europe, most likely through conversion. The phylogenetic nesting patterns suggest that the most frequent of the Ashkenazi mtDNA lineages were assimilated in Western Europe, ~2 ka or slightly earlier. Some in particular, including N1b2, M1a1b, K1a9 and perhaps even the major K1a1b1, point to a north Mediterranean source. It seems likely that the major founders were the result of the earliest and presumably most profound wave of founder effects, from the Mediterranean northwards into central Europe, and that most of the minor founders were assimilated in west/central Europe within the last 1,500 years. The sharing of rarer lineages with Eastern European populations may indicate further assimilation in some cases, but can often be explained by exchange via intermarriage in the reverse direction.

The Ashkenazim therefore resemble Jewish communities in Eastern Africa and India, and possibly also others across the Near East, Caucasus and Central Asia, which also carry a substantial fraction of maternal lineages from their ‘host’ communities^11^,^25^. Despite widely differing interpretations of autosomal data, these results in fact fit well with genome-wide studies, which imply a significant European component, with particularly close relationships to Italians^3^,^4^,^6^,^7^. As might be expected from the autosomal picture, Y-chromosome studies generally show the opposite trend to mtDNA (with a predominantly Near Eastern source) with the exception of the large fraction of European ancestry seen in Ashkenazi Levites^22^.

Evidence for haplotype sharing with non-Ashkenazi Jews for each of the three main haplogroup K founders may imply a partial common ancestry in Mediterranean Europe for Ashkenazi and Spanish-exile Sephardic Jews, but may also, at least in part, be due to subsequent gene flow, especially into Bulgaria and Turkey, both of which witnessed substantial immigration from Ashkenazi communities in the fourteenth and fifteenth centuries. Gene flow could have been substantial in some cases—ongoing intermarriage is likely when these communities began living in closer proximity after the Spanish exile^6^. A partial common ancestry for all European Jews—both Ashkenazi and Sephardic—is again strongly supported by the autosomal results^3^,^4^.

Jewish communities were already spread across the Graeco-Roman and Persian world >2,000 years ago. It is thought that a substantial Jewish community was present in Rome from at least the mid-second century BCE, maintaining links to Jerusalem and numbering 30,000–50,000 by the first half of the first century CE^15^. By the end of the first millennium CE, Ashkenazi communities were historically visible along the Rhine valley in Germany^33^. After the wave of expulsions in Western Europe during the fifteenth century, they began to disperse once more, into Eastern Europe^33^.

These analyses suggest that the first major wave of assimilation probably took place in Mediterranean Europe, most likely in the Italian peninsula ~2 ka, with substantial further assimilation of minor founders in west/central Europe. There is less evidence for assimilation in Eastern Europe, and almost none for a source in the North Caucasus/Chuvashia, as would be predicted by the Khazar hypothesis^8^,^9^—rather, the results show strong genetic continuities between west and east European Ashkenazi communities^10^, albeit with gradual clines of frequency of founders between east and west^1^,^2^ (Supplementary Note 2).

There is surprisingly little evidence for any significant founder event from the Near East. Fewer than 10% of the Ashkenazi mtDNAs can be assigned to a Near Eastern source with any confidence, and these are found at very low frequencies (Fig. 2). The most frequent, belonging to HV1b2, R0a1a and U7, are found at only ~3, 2 and 1% respectively. All are widespread across Ashkenazi communities, and might conceivably be relicts of early Levantine founders, but it seems likely that other more minor Near Eastern lineages are the result of more recent gene flow into the Ashkenazim.

The age estimates for the European founders might suggest (very tentatively, given the imprecision with present data) that these ancestral Jewish populations harboring haplogroup K and especially N1b2 may have had an origin in the first millennium BCE, rather than in the wake of the destruction of the Jerusalem Temple in 70 CE. In fact, some scholars have argued from historical evidence that the large-scale expansion of Judaism throughout the Mediterranean in the Hellenistic period was primarily the result of proselytism and mass-conversion, especially amongst women^9^. We anticipate that a combination of large-scale mitogenome and whole Y-chromosome analysis, complementing full human genome sequencing, will be able to address this question in much finer detail in the near future.

Despite the potential of genomic studies, the particular value of full-mitogenome sequencing should be stressed, as some studies dismissed the value of uniparental markers because of the impact of drift in the Ashkenazim^6^. In fact, the reverse may be the case: autosomal studies may be confounded by drift whereas the fine genealogical resolution of full mitogenomes, given sufficient sampling, can provide a detailed reconstruction of the history of Ashkenazi women. The mtDNA genealogy may even be considered to have particular relevance due to the matrilineal inheritance found in Judaism since at least ~200 CE and possibly several centuries earlier, helping to ‘fix’ incoming lineages from converts within the Ashkenazi community after this time. With sufficient resolution, a detailed genealogical history for every maternal lineage in the Ashkenazim is now within reach; in fact, it should soon be possible to reconstruct the outlines of the entire dispersal history of each community.

## Methods

### Samples and analysis of mtDNA sequence variation

Although there is a growing database of whole mitogenomes, almost all those from haplogroup U8 are from Europeans or individuals of European (predominantly west European) ancestry. Yet evidence from the Near East is critical in drawing up a meaningful picture of European (and wider west Eurasian) demographic prehistory. We therefore selected 67 predominantly Near Eastern haplogroup K samples (identified by full control-region sequencing of 111 haplogroup K samples) for mitogenome sequencing, plus five samples belonging to non-K U8 and two from Italy potentially belonging to N1b2 (Supplementary Data 1). We collected samples with the appropriate informed consent of the subjects and the work was approved by the University of Leeds, Faculty of Biological Sciences Ethics Committee, the Ethics Committee for Clinical Experimentation at the University of Pavia, and the Western Institution Review Board (WIRB), Olympia, WA, USA. We sequenced them using Sanger sequencing^30^,^34^ and, to maximize the number of samples, we performed a phylogenetic analysis alongside 884 published U8 sequences (a total of 909 belonging to haplogroup K) (Supplementary Data 1) and four haplogroup N outgroup sequences, using Network 4.6 software and the reduced-median algorithm^35^. We then constructed a putative most-parsimonious tree of the 956 U8 sequences by hand from the network, following PhyloTree^36^ for known subclades. We used mtDNA-GeneSyn^37^ to convert files. As there are a number of extremely variable sites in K1 (positions 195 and 16,093 in particular), we confirmed the overall topology by running networks of coding-region data only. We performed similar analyses for haplogroups H, J and T, and for N1b we augmented our previously published tree^24^.

### Age estimates and phylogeographic distribution

We estimated coalescence times of clades, using the *ρ* statistic and ML^38^,^39^, with Bayesian estimations for mitogenomes using BEAST^40^. For the *ρ* statistic and ML, we corrected for purifying selection using the calculator we developed previously (Supplementary Data 4)^38^. We defined some sub-haplogroups to be *a priori* monophyletic in the analysis (U8, U8a, U8b, K, K1, K1a, K1b, K1c, K2, K2a and K2b) and assumed a generation time of 25 years^41^. We also obtained Bayesian skyline plots^42^,^43^,^44^ to estimate ‘haplogroup-effective’ population sizes associated with U8 over time, and estimated the period of maximum growth^39^.

For a broader overview of the diversity and geographic distribution of lineages, we also compiled 1,917 haplogroup K HVS-I (hypervariable sequence I) sequences (in the range 16,051–16,400), 87 from U8a and 52 from U8b1 (from Europe, the Near East and North Africa, from a total database of 33,127 HVS-I sequences) (Supplementary Tables S1 and S2). We displayed frequency and diversity distributions of haplogroups K, U8a1 and U8b1 sequences, identified from their motifs in the HVS-I database, on interpolation maps using Surfer. For the frequency analyses, we analysed the data at the level of published regional populations; for the diversity analyses we aggregated them into broader areas, as described in Supplementary Table S2. For the analyses of other Ashkenazi lineages we compared 836 published control-region sequences^1^,^2^,^11^ with available Ashkenazi whole mitogenomes and the global mitogenome database available on GenBank, in order to assign the Ashkenazi control-region lineages to subclades. For geographic distributions, we supplemented and checked this information against a database of control-region data (38,244 records from west Eurasia, Central Asia and North Africa).

## Author contributions

M.B.R., L.P. and P.S. devised and supervised the project, M.D.C., J.B.P., M.C. and A.O. carried out the laboratory work, M.D.C., J.B.P., M.P., V.F., P.S., L.P. and M.B.R. carried out the data analyses, M.D.C., J.B.P., P.S., L.P. and M.B.R. wrote the text, M.P., A.O., A.A., U.A.P., S.R., ON., J.H., S.R.W., K.K.E., M.C. and V.M. discussed the results and helped to revise the text.

## Additional information

**How to cite this article:** Costa, M.D. *et al.* A substantial prehistoric European ancestry amongst Ashkenazi maternal lineages. *Nat. Commun.* 4:2543 doi: 10.1038/ncomms3543 (2013).

**Data access:** Sequence data have been deposited in GenBank nucleotide core database under accession numbers JX273243 to JX273297, KC878709 to KC878725 and KF297808 to KF297809.

## Supplementary Material

Supplementary Tables, Figures, Notes and References Supplementary Figures S1-S10, Supplementary Tables S1-S9, Supplementary Notes 1-3 and Supplementary References

Supplementary Data 1 Phylogenetic tree of haplogroup U8 mitogenome sequences

Supplementary Data 2 Age estimates using rho (ρ), maximum likelihood and Bayesian estimation for haplogroup U8 and its subclades

Supplementary Data 3 U8 mitogenomes included in the phylogenetic and phylogeographic analysis

Supplementary Data 4 Calculator for converting mtDNA ρ and ML values to age estimates
