# Supplementary material for: A substantial prehistoric European ancestry amongst Ashkenazi maternal lineages
Source: Nat Commun. 2013 Oct 8;4:2543. doi: 10.1038/ncomms3543 (PMC3806353; doi:10.1038/ncomms3543)
Supplement: Supplementary Tables, Figures, Notes and References — Supplementary Figures S1-S10, Supplementary Tables S1-S9, Supplementary Notes 1-3 and Supplementary References [file ncomms3543-s1.pdf]

Supplementary Figures

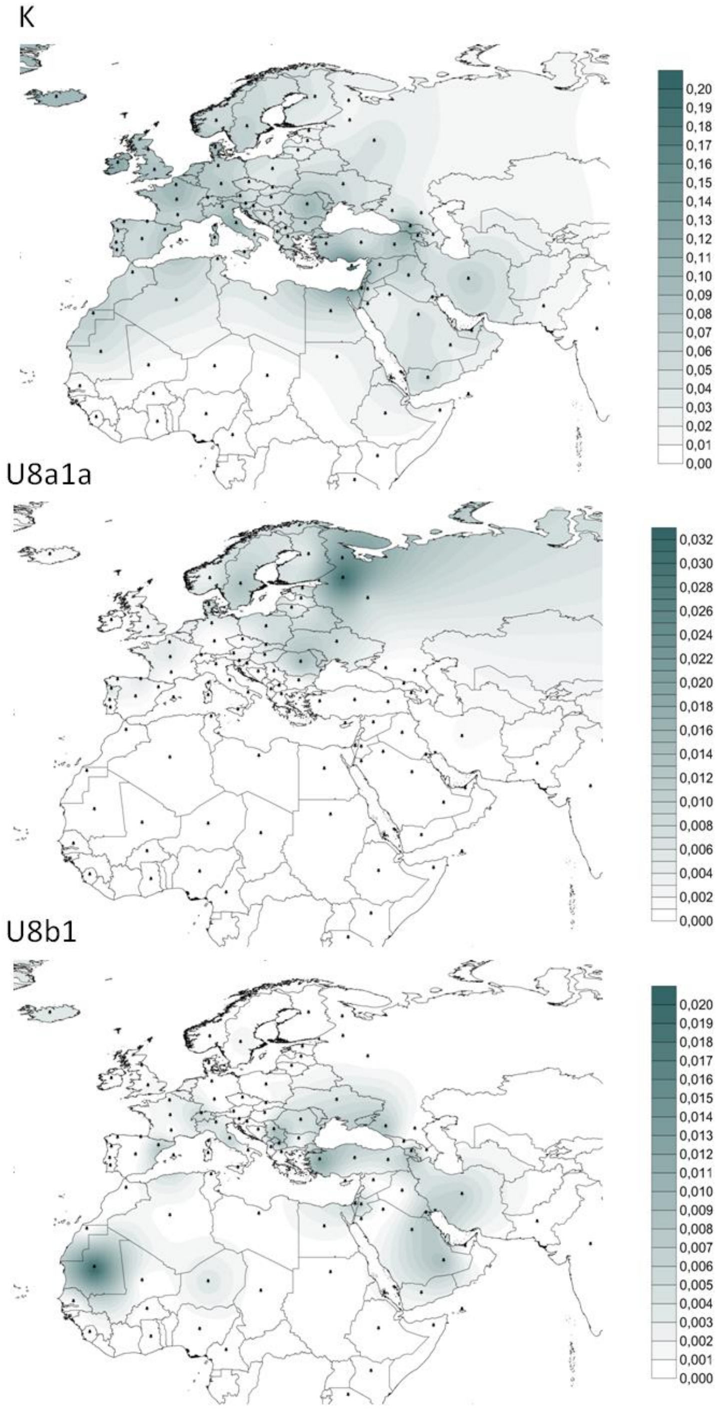

Supplementary Figure S1. Frequency distribution maps for mtDNA haplogroups K, U8a1a and U8b1. Maps created using Surfer and based on the data presented in Supplementary Table S2. Dots represent sampling locations used for the spatial analysis.

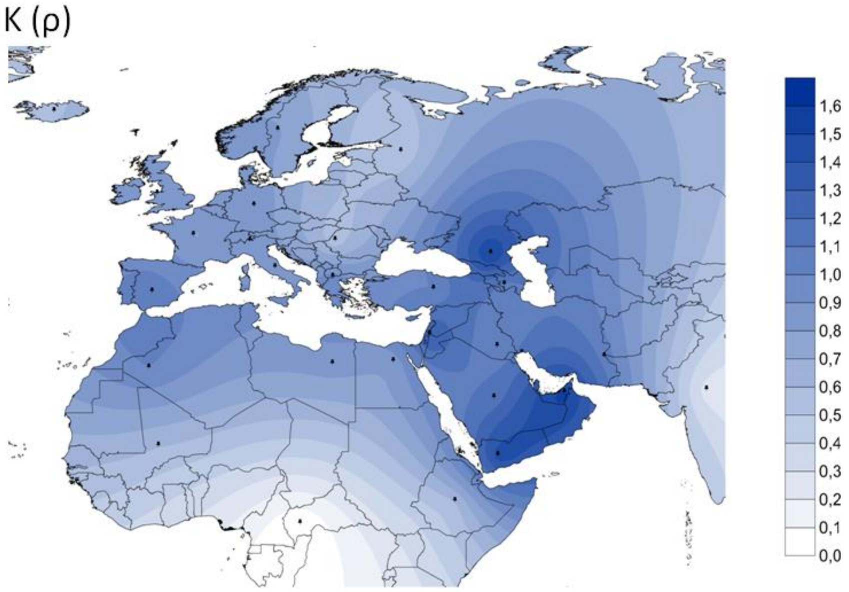

Supplementary Figure S2. Distribution map of the diversity measure ρ for haplogroup K. Map created using Surfer. Dots represent sampling locations used for the spatial analysis.

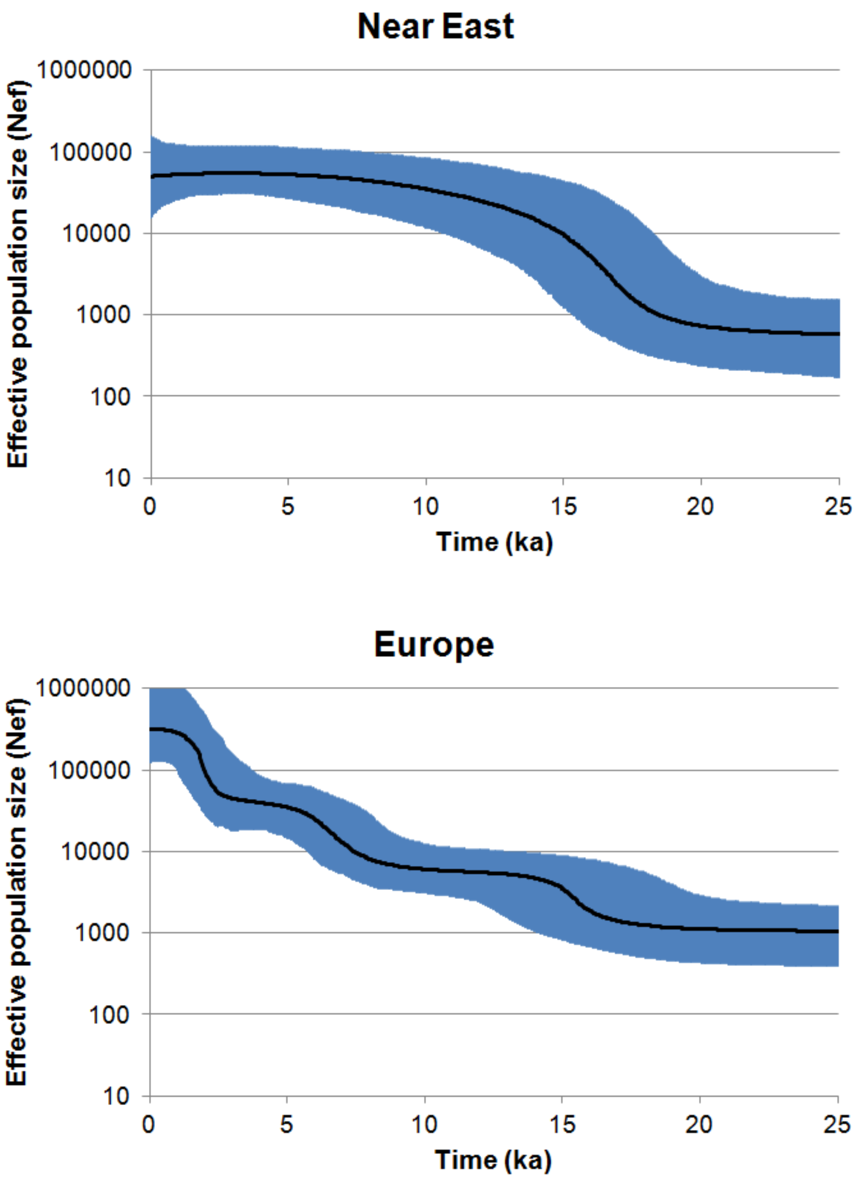

Supplementary Figure S3. Bayesian skyline plots based on haplogroup U8 mitogenome data in the Near East/Caucasus and Europe. The hypothetical effective population size is indicated (on a logarithmic scale) through time (ka). The sample sizes were 106 and 474 respectively. The posterior effective population size through time is represented by the black line. The blue region represents the 95% confidence interval.

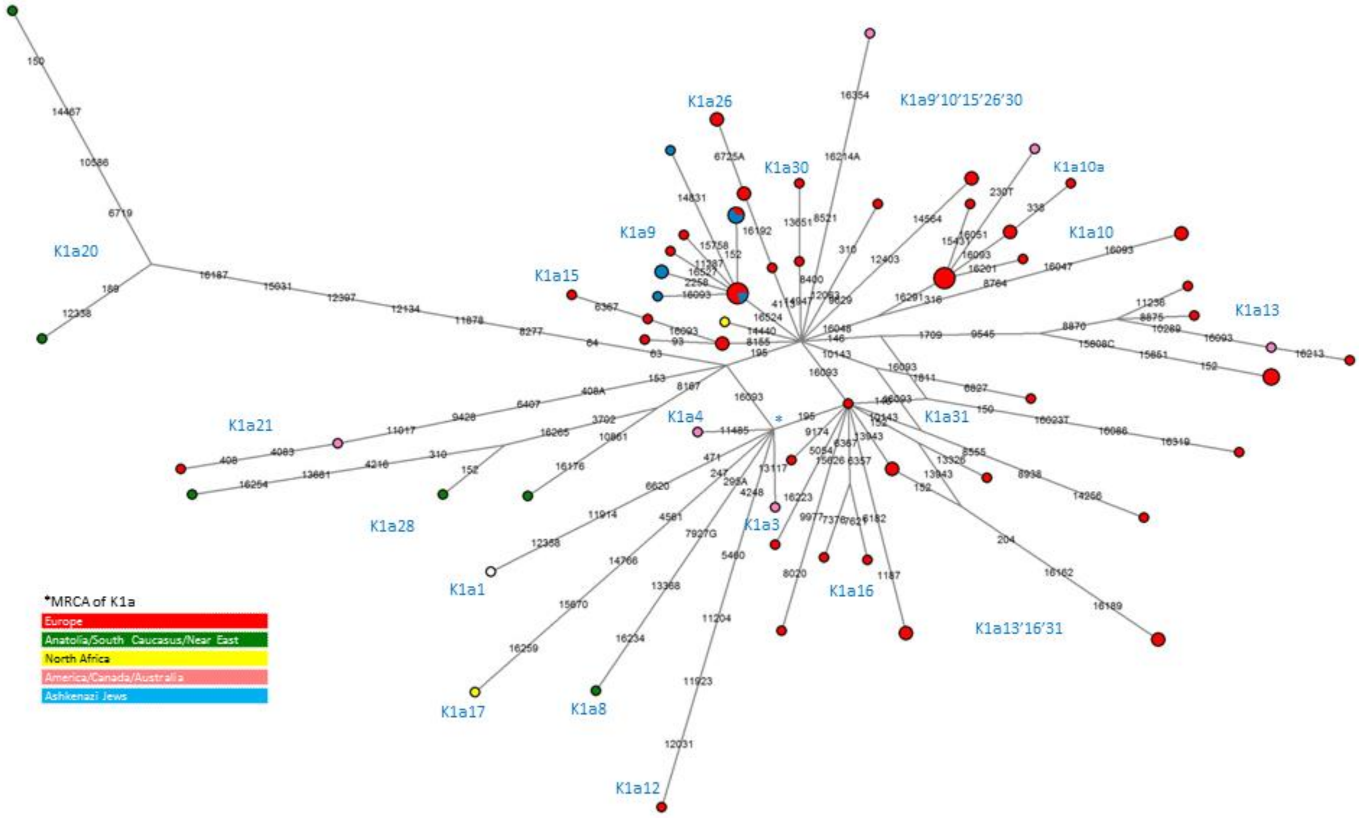

Supplementary Figure S4. Reduced-median network of haplogroup K1a9, in the context of putative clade K1a9'10'15'26'30. The network is rooted with several additional lineages from K1a. The tree in Supplementary Data 1 is resolved by assuming transitions first at position 16093 and then at position 195, with K1a20, K1a28 and K1a30 branching off earlier. The tree could also be resolved by assuming mutation at position 195 first, then at 16093, in which case K1a13, K1a16 and K1a31 would branch off earlier, rather than forming a distinct terminal subclade, K1a13'16'31. The latter resolution would considerably extend the European ancestry of the K1a9 lineage.

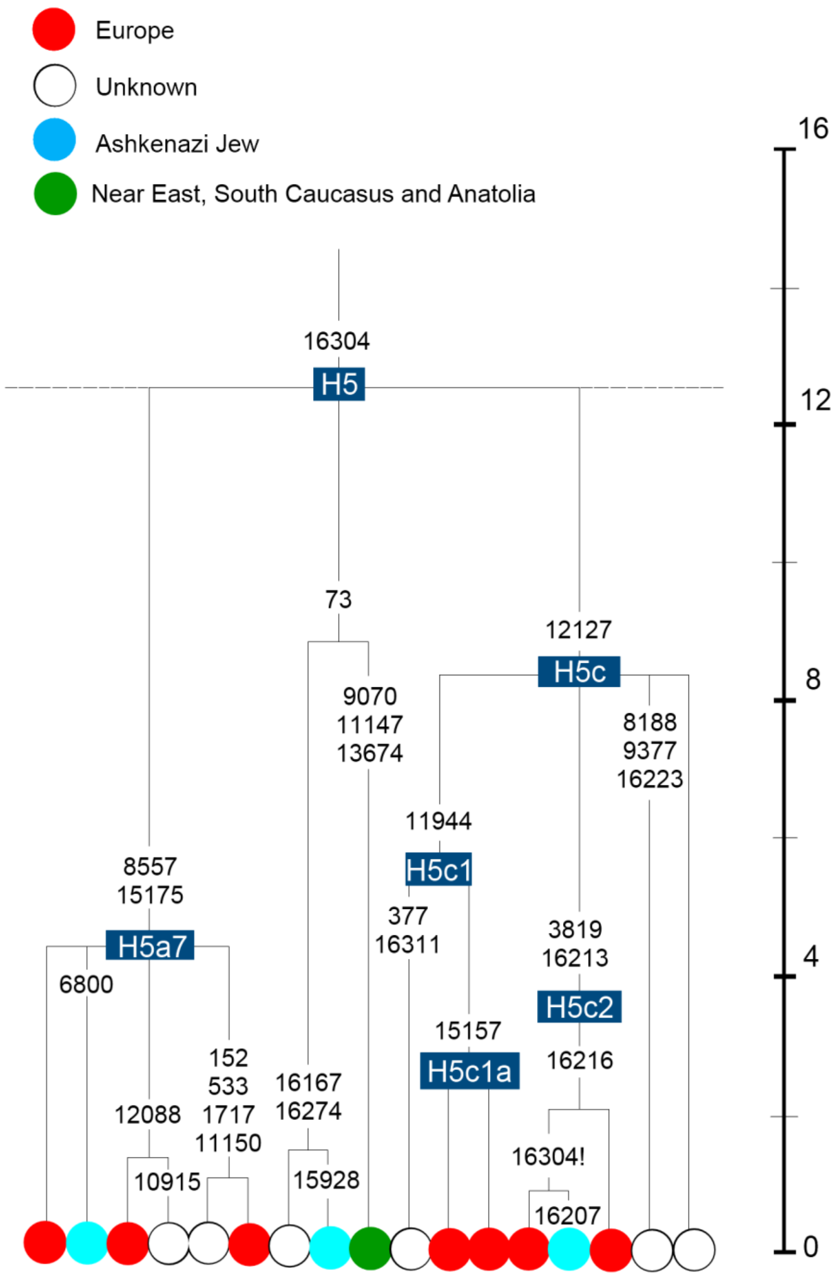

Supplementary Figure S5. Phylogenetic tree of Ashkenazi founders within haplogroup H5. Time scale (ka) based on ML estimations for mitogenome sequences.

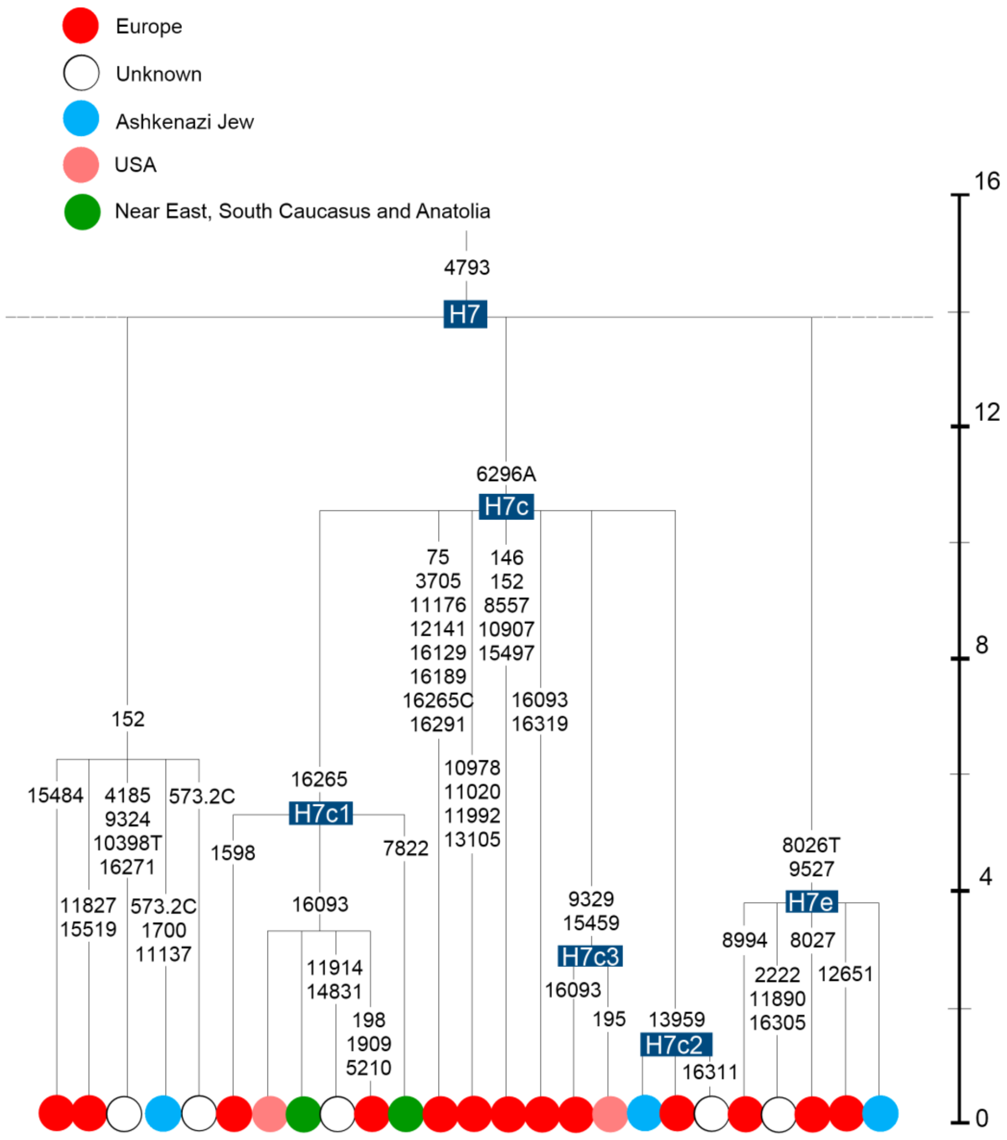

Supplementary Figure S6. Phylogenetic tree of Ashkenazi founders within haplogroup H7. Time scale (ka) based on ML estimations for mitogenome sequences.

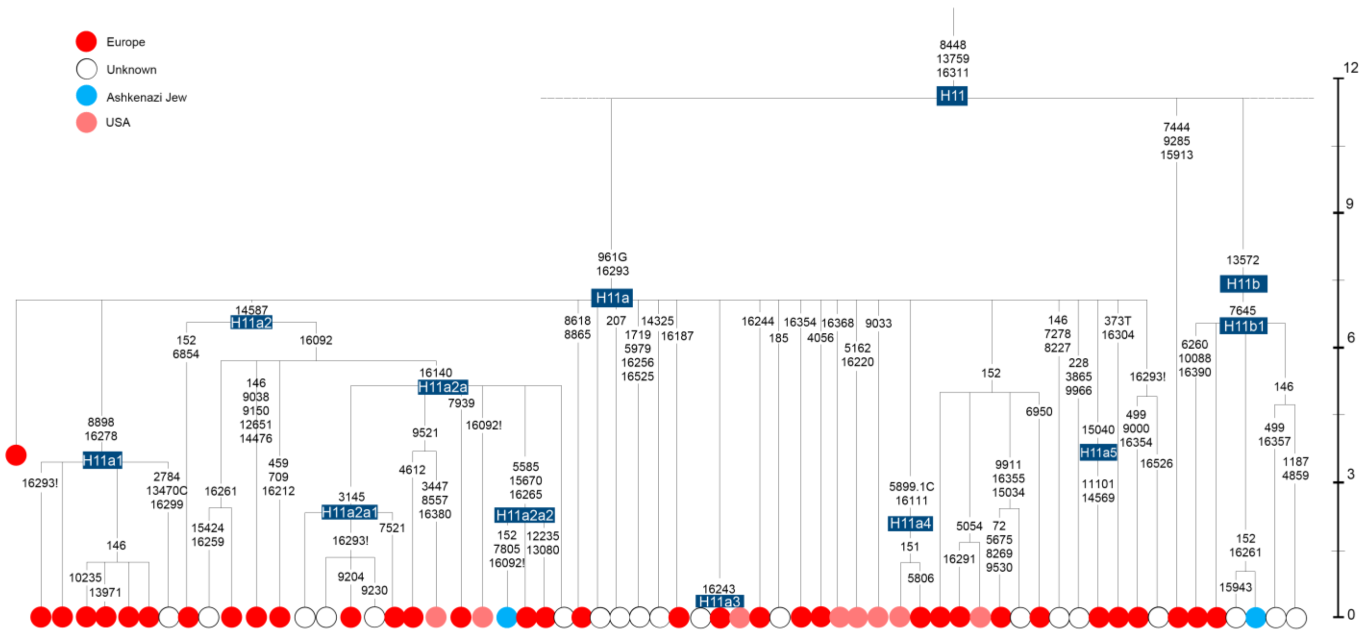

Supplementary Figure S7. Phylogenetic tree of Ashkenazi founders within haplogroup H11. A Bronze Age European lineage is shown with a red circle[29]. Time scale (ka) based on ML estimations for mitogenome sequences.

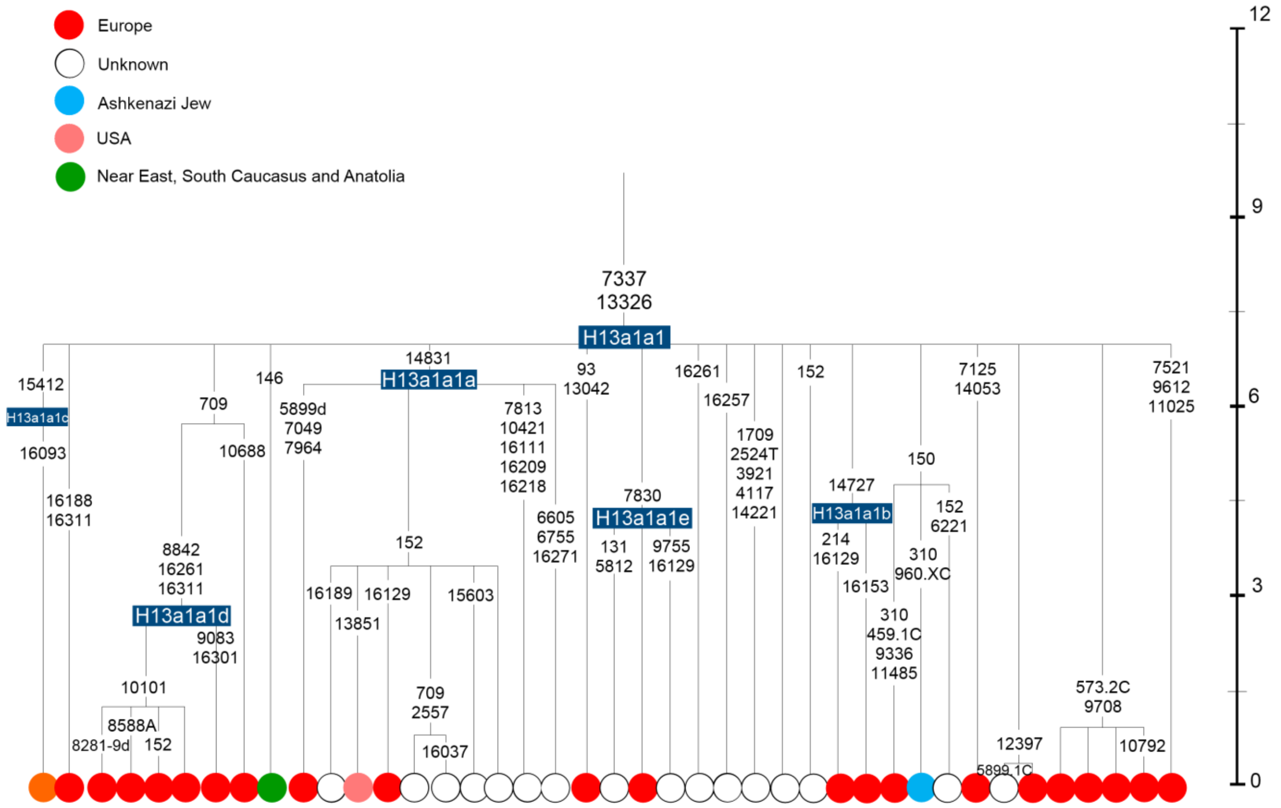

Supplementary Figure S8. Phylogenetic tree of Ashkenazi founder within haplogroup H13a1a1. Time scale (ka) based on ML estimations for mitogenome sequences.

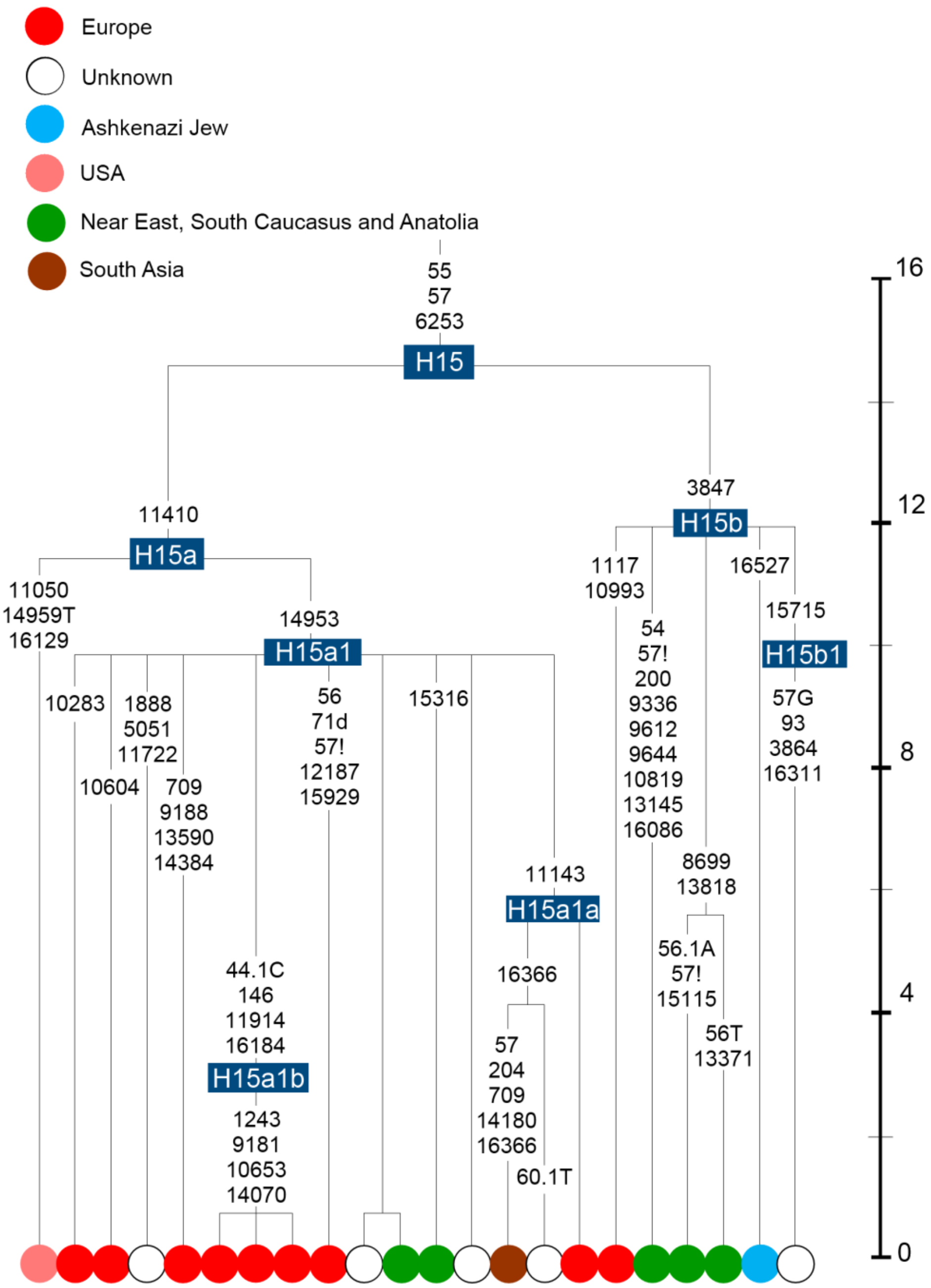

Supplementary Figure S9. Phylogenetic tree of Ashkenazi founder within haplogroup H15. Time scale (ka) based on ML estimations for mitogenome sequences.

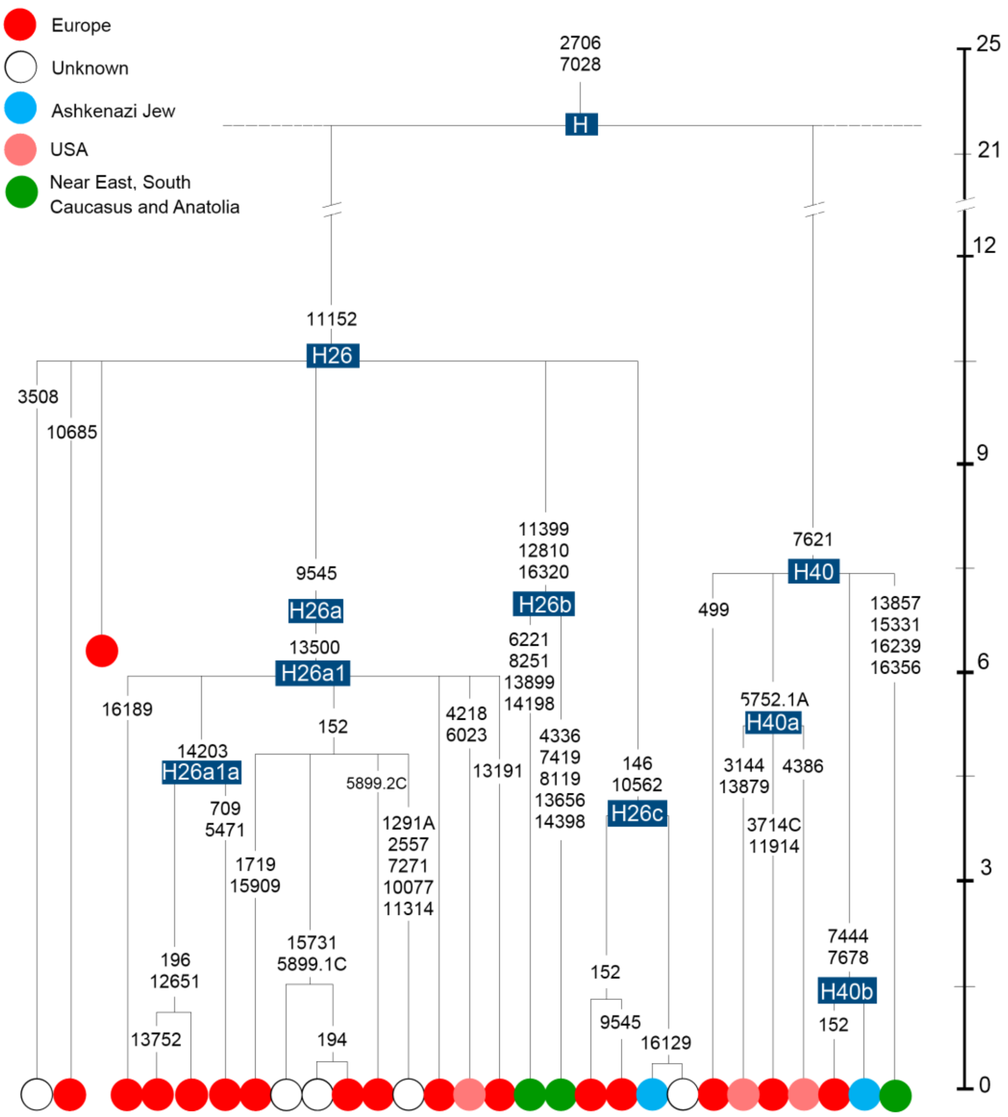

Supplementary Figure S10. Phylogenetic tree of Ashkenazi founders within haplogroups H26 and H40. A Neolithic European lineage is shown with a red circle[29]. Time scale (ka) based on ML estimations for mitogenome sequences.

Supplementary Tables

Supplementary Table S1. Frequencies of mtDNA haplogroups K, U8a1 and U8b1.

Geographic Area	Region	N	U8a1a	U8b1	K
AFRICA	Algeria	47	—	—	0.043
	Libya	398	—	—	0.035
	Tunisia	551	—	—	0.038
	Western Sahara	56	—	—	0.071
	Egypt	594	—	0.002	0.047
	Ethiopia	347	—	—	0.017
	Morocco	1014	—	—	0.045
	Sudan	178	—	—	0.017
	Somalia	148	—	—	0.007
	Kenya	188	—	—	0.005
	Tanzania	100	—	—	0.010
	Burkina Faso	137	—	—	—
	Cameroon	863	—	—	0.001
	Chad	141	—	—	—
	Ghana	238	—	—	—
	Mali	305	—	—	0.006
	Mauritania	94	—	0.021	0.042
	Niger	239	—	0.004	—
	Nigeria	1457	—	—	—
	Senegal	280	—	—	—
	Sierra Leone	276	—	—	—
EUROPE	Austria	374	0.003	—	0.080
	Bulgaria	138	—	—	0.065
	Croatia	204	—	—	0.054
	Cyprus	91	—	—	0.209
	Czech Republic	175	—	—	0.034
	Denmark	201	0.010	—	0.100
	Estonia	266	0.008	—	0.023
	Finland	661	0.005	—	0.041
	France				
	North	435	0.005	0.002	0.101
	Central	631	0.005	0.002	0.097
	South	166	—	—	0.048
	Corsica	53	—	—	0.094
	Germany				

	North	1741	0.002	—	0.062
	South	100	—	—	0.080
	Bosnia	144	—	—	0.042
	Greece	706	—	—	0.054
	Hungary	211	0.005	—	0.047
	Iceland	985	0.001	0.004	0.098
	Italy				
	North	830	—	0.004	0.075
	Central	267	—	0.007	0.116
	South	136	—	—	0.059
	Sardinia	341	—	—	0.062
	Sicily	138	—	—	0.051
	Latvia	413	—	—	0.024
	Lithuania	343	0.006	—	0.023
	Ireland	266	—	—	0.120
	Poland	882	0.007	0.001	0.039
	Portugal				
	North	271	0.007	—	0.048
	Central	342	—	—	0.067
	South	519	—	—	0.067
	Romania	600	0.015	0.005	0.107
	Russia				
	Karelia	594	0.024	—	0.017
	Novgorod Oblast	157	0.032	—	0.019
	Central, Volga Finns and Chuvash	359	0.008	—	0.047
	Slovakia	710	0.006	—	0.038
	Slovenia	232	0.004	—	0.060
	Spain				
	Basque Country	568	0.005	—	0.048
	Northwestern	526	—	—	0.076
	Northeastern	126	—	0.008	0.079
	Central–South	586	0.002	—	0.056
	Balearic Islands	256	—	0.008	0.105
	Sweden	634	0.014	0.002	0.069
	Switzerland	153	—	0.007	0.052
	British Isles				
	North	1691	0.002	—	0.076
	South	2216	0.003	0.001	0.081
	Norway	305	0.003	—	0.049
	Albania	102	—	—	0.039
	Macedonia	308	—	0.010	0.055
	Serbia	104	—	0.010	0.038
**NORTH CAUCASUS**	West (Kabardia, Karachay-Cherkess, Northwest Caucasus)	116	—	0.009	0.034

	East (Avars, Lezginians, Ruthulians, North Ossetia)	334	—	—	0.027
SOUTH & SOUTHWEST ASIA	Armenia	191	—	0.010	0.073
	Azerbaijan	48	—	—	0.021
	Georgia, Abkhazia	101	—	—	0.139
	Turkey				
	East (with Kurds)	222	—	0.005	0.086
	Central	202	—	0.005	0.035
	West	155	—	0.013	0.077
	Syria	141	—	—	0.064
	Israel-Palestine	525	—	0.008	0.118
	Jordan	143	—	0.007	0.035
	Iraq	167	—	—	0.060
	Kuwait	381	—	0.008	0.026
	Dubai	249	—	—	0.044
	Saudi Arabia				
	North	42	—	—	0.024
	South	106	—	0.009	0.028
	Central	332	—	0.006	0.048
	West (with Bedouin)	160	—	—	0.031
	Yemen	387	—	0.003	0.057
	Yemen, Socotra	65	—	—	—
	Iran	738	0.001	0.007	0.072
	Pakistan	189	—	—	0.016
	India	2649	—	—	0.001

Supplementary Table S2. Haplogroup K diversity measures (ρ and π) based on regional HVS-I data.

Geographic Region	No. of K haplotypes	ρ	SD	π
Africa Cyrenaica	9	0.857	0.295	1.582
East Africa	5	0.583	0.300	1.867
Northeast Africa	13	0.821	0.283	1.537
Northwest Africa	28	1.000	0.362	1.882
West Africa	2	0.600	0.600	0.667
Central Africa	1	—	—	—
**Africa Total**	**58**			
Alps	17	0.842	0.291	1.539
British Isles	73	0.903	0.242	1.687
Iceland	9	0.577	0.379	0.876
Mediterranean Central	52	0.876	0.246	1.647
Mediterranean East	31	0.961	0.223	1.776
Mediterranean West	58	1.074	0.334	1.915
North Central Europe	48	0.871	0.217	1.650
North Eastern Europe	24	0.512	0.136	1.352
North Western Europe	33	0.788	0.220	1.400
Scandinavia	30	0.814	0.208	1.281
Southeast Europe	37	0.538	0.171	1.330
**Europe Total**	**412**			
North Caucasus	9	1.533	0.562	2.615
South Caucasus	9	1.044	0.426	1.828
**Caucasus Total**	**18**			
Anatolia	18	1.000	0.360	1.731
Iran/Pakistan	34	1.057	0.279	1.890
Levant	21	1.330	0.434	2.293
**Near East Total**	**73**			
Central Arabian Peninsula	8	1.136	0.475	1.909
Eastern Arabian Peninsula	11	1.546	0.828	1.829
Southwest Arabian Peninsula	11	1.455	0.591	2.268
Mesopotamia	4	1.000	0.436	1.356
**Arabia/Mesopotamia Total**	**34**			
India	2	0.250	0.250	0.500

Supplementary Table S3. Age estimates using rho (ρ) for haplogroup U8 and its major subclades dated separately in the Near East and Europe. Ages and 95% confidence intervals (CI) in thousands of years.

Clade	EUROPE		NEAR EAST	
	Age	CI	Age	CI
**K**	26,200	[18,100–34,700]	28,300	[18,400–38,600]
**K1**	20,800	[15,400–26,300]	22,600	[16,400–28,900]
**K1a**	17,000	[12,600–21,500]	19,500	[16,000–23,000]
**K1a4**	16,900	[9800–24,300]	20,600	[15,400–25,900]
**K1b1**	25,800	[13,800–38,400]	13,400	[3300–24,000]
**K2**	18,700	[11,000–26,700]	17,600	[7800–27,900]
**U8**	50,300	[32,300–69,100]	53,500	[33,800–74,200]
**U8b**	41,500	[25,800–58,100]	43,900	[27,800–60,900]

Supplementary Table S4. Age estimates using rho (ρ) and ML for haplogroup HV1b and its subclades. Ages and 95% confidence intervals (CI) in thousands of years.

Clade	ρ (complete sequences)		ρ (synonymous)		ML	
	Age	CI	Age	CI	Age	CI
**HV1b**	17,200	[10,200–24,400]	14,200	[5000–23,300]	18,200	[10,200–26,600]
**HV1b+152**	12,500	[5600–19,700]	12,300	[1000–23,600]	14,900	[8600–21,400]
**HV1b2**	2100	[700–3500]	2900	[100–5700]	2200	[800–3600]
**HV1b3**	19,000	[8600–30,000]	18,400	[3000–33,800]	12,300	[6500–18,200]
**HV1b1**	10,300	[4800–15,900]	9900	[1700–18,000]	8700	[4500–13,100]
**HV1b1b**	6600	[1100–12,200]	5200	[0–12,500]	5900	[1800–10,100]
**HV1b1a**	8800	[1900–16,000]	10,500	[0–23,100]	5900	[2100 –9900]

Supplementary Table S5. Age estimates using rho (ρ) and ML for haplogroup N1b and its subclades. Ages and 95% confidence intervals (CI) in thousands of years.

Clade	ρ (complete sequences)		ρ (synonymous)		ML	
	Age	CI	Age	CI	Age	CI
**N1b**	21,200	[13,600–29,100]	25,000	[9000–40,900]	19,100	[13,200–25,100]
**N1b1**	13,900	[10,800–17,000]	12,800	[7300–18,200]	13,500	[6600–20,600]
**N1b1b**	11,600	[7600–15,800]	16,700	[8800–24,700]	11,600	[8100–15,200]
**N1b1c**	8700	[3900–13,400]	4000	[0–9400]	9500	[4700–14,400]
**N1b1+16129**	15,000	[8700–21,600]	15,700	[5100–26,400]	12,600	[9200–16,000]
**N1b1d**	13,400	[5200–21,900]	13,100	[0–26,700]	8700	[4700–12,700]
**N1b2**	4900	[100–9800]	9300	[0–23,200]	5000	[1800–8300]
**N1b2+14581**	2400	[700–4100]	2800	[0–6500]	2100	[700–3500]

Supplementary Table S6. Summary of distributions of the main haplogroups in the Ashkenazi mtDNA control-region data[1,2,11,45]. "HV" includes HV1, a single HV2 lineage, two HV13 lineages and five putative HV5 lineages; "U" includes U1, U2, U3, U4, U6 and a single U8b1 lineage; "others" include single N1c, N9a and two putative K1a4 lineages. Note that the British data are excluded from the Western European totals because British Ashkenazi Jews have a predominantly Eastern European ancestry, as is evident in the haplogroup frequencies.

Region	H	HV	HV0	I	J	K1a1b1a	K1a9	K2a	L2a1l	M1a1b	N1b2	R0a1a	T	U	U5	U7	W	X	M/A	Others
**Eastern Europe**	24.3	5.8	4.3	0.9	7.5	18.7	6.0	6.0	1.5	0.3	8.8	1.8	4.3	2.3	2.3	0.9	2.0	0.6	0.9	0.6
**Czech/Austria/ Hungary**	30.4	1.8	6.9	1.4	3.2	21.7	5.1	7.4	0.9	—	9.7	—	4.6	1.4	1.8	—	2.3	0.5	0.5	0.5
**Baltic**	30.8	7.7	5.1	—	—	15.4	7.7	5.1	—	2.6	15.4	7.7	—	2.6	—	—	—	—	—	—
**Poland**	13.5	7.6	2.9	1.8	10.6	25.9	4.7	7.6	2.9	0.6	5.9	1.2	3.5	4.1	1.2	1.8	2.4	0.6	1.2	—
**Russia/Belarus**	19.2	12.3	—	—	16.4	5.5	4.1	5.5	1.4	—	8.2	5.5	8.2	1.4	5.5	—	4.1	—	1.4	1.4
**Romania/Moldavia**	24.5	6.1	1.0	—	10.2	16.3	9.2	4.1	2.0	—	10.2	1.0	3.1	1.0	3.1	1.0	1.0	2.0	2.0	2.0
**Ukraine**	35.2	5.6	9.3	—	3.7	9.3	9.3	—	—	—	7.4	3.7	5.6	3.7	3.7	3.7	—	—	—	—
**Western Europe**	15.2	—	1.9	—	4.8	34.3	8.6	1.9	1.9	3.8	14.3	2.9	4.8	1.0	—	1.9	—	1.9	1.0	—
**France**	14.3	—	—	—	4.1	30.6	6.1	4.1	2.0	6.1	16.3	2.0	6.1	2.0	—	2.0	—	2.0	2.0	—
**Netherlands/ Germany/ Switzerland**	16.1	—	3.6	—	5.4	37.5	10.7	—	1.8	1.8	12.5	3.6	3.6	—	—	1.8	—	1.8	—	—
**Great Britain**	21.3	5.0	5.0	6.3	3.8	13.8	6.3	3.8	—	—	6.3	2.5	8.8	11.3	2.5	1.3	—	1.3	1.3	—
**Total**	22.8	5.0	4.1	1.3	6.8	20.2	6.3	5.3	1.4	0.7	9.2	2.0	4.8	3.0	2.0	1.1	1.6	0.8	1.0	0.5

Supplementary Table S7. List of 29 Ashkenazi mitogenome haplogroup H sequences used in the analysis. Three Sephardic and one B'nei Israel haplogroup H sequences are also shown.

Accession numbers	Control-region variants motif	Haplogroup	Notes (*e.g.* presence in prehistoric central European remains[29])
**JQ703788, JQ703655**	16172C-16192T-16456ª-207A	H1aj1	*H1 found in Early Neolithic remains*
**JQ704370**	CRS	H1as	*H1 found in Early Neolithic remains*
**JQ703268, JQ704894**	16080G-16189C-16356C-16360T-183G	H1b2a1	*Subclade found mainly in central/Eastern Europe*
**EU148452, EU262984, JQ703137, JQ705236**	16114T; one has 16126C	H1e4a	*H1e found in Early Neolithic remains*
**JQ701904**	152C-16319A	H2a2a2	*H2 found in Bronze Age remains*
**JQ704133, JQ703179**	16222T-16299G-146C; one has 200G	H3p	*H3 found in mid-Neolithic remains*
**JQ705703**	195C-513A	H4a1a3a	*H4 found in Late Neolithic Corded Ware remains*
**JQ704150**	16304C-456T	H5a7	*H5a found in Late Neolithic Bell Beaker remains*
**HQ663853**	16167T-16274A-16304C-73G-456T	H5-73G	*Sample designated only as "Jewish"*<br>*H5 found in Early Neolithic remains*
**JF790201**	16207G-16213A-16216G-456T	H5c2	*H5 found in Early Neolithic remains*
**JQ702509**	16311C-16362C-16482G-239C	H6a1a1a	*H6a1a found in Late Neolithic Corded Ware remains; Near Eastern subclade H6a1a1*
**HQ730608**	16362C-16482G-239C	H6a1a3	*H6a1a in Corded Ware remains*
**GU390313, HM015668**	16362C-16482G-239C	H6a1a5	*H6a1a in Corded Ware remains*
**JQ703313**	152C-573.1CC	H7-152C	*H7 found in mid-Neolithic and Bronze Age remains*
**JQ703601**	CRS	H7c2	*H7 found in mid-Neolithic and Bronze Age remains*
**HQ267514**	CRS	H7e	*H7 found in mid-Neolithic and Early Bronze Age remains*
**JQ704427**	16092C-16140C-16293G-16311C-152C-195C	H11a2a2	*H11a found in Early Bronze Age remains*
**JQ704081**	16261T-16311C-152C-195C	H11b1	*H11 found in Early Bronze Age remains*
**JQ701818**	150T-310C	H13a1a1	*H13a1a2 found in Late Neolithic Bell Beaker remains*
**JQ704870**	CRS-55C-57C-16527T	H15b	*H15b may be Near Eastern*
**JQ704110**	146C-16129A	H26c	*H26 found in Early Neolithic remains*
**JQ702933**	CRS	H40b	*Source unresolved*
**EF556184**	CRS	H1p	*Turkish (Sephardic) Jew*
**EF556189**	CRS	H25	*Bulgarian Jew – Sephardic (unresolved – direct from root)*
**JQ703478**	16093C	H56	*Sephardic Jew from Spain (European clade)*
**EF556150**	183G-709A	H13a2a	*B'nei Israel-Jew (Indian/Near Eastern clade)*

Supplementary Table S8. Age estimates using rho (ρ) and ML for haplogroup H and its subclades. Ages and 95% confidence intervals (CI) in thousands of years. Subclades carried by Ashkenazim are shown in pale blue.

Clade	ρ				ML	
	Whole mtDNA		Synonymous		Whole mtDNA	
	Age	CI	Age	CI	Age	CI
H1	11,000	9100–12,800	10,100	8000–12,300	11,800	10,300–13,300
H1as	7100	2400–12,000	4500	0–11,500	7600	2600–12,800
H1as1	7000	1000–13,200	0	0	4800	700–9100
H1as2	1700	0–4100	2600	0–7800	2300	0–6600
H1aj	16,200	5300–27,600	1600	0–4700	10,200	5600–14,800
H1aj1	2600	2500–24,900	0	0	2200	0–5200
H1b2	7100	1500–12,900	0	0	5300	2200–8500
H1b2a	1300	0–3800	0	0	1100	0–2300
H1e	11,400	7000–16,000	18,200	6000–30,300	10,900	8400–13,300
H1e4	3200	0–7000	0	0	3400	0–7600
H1e4a	1500	0–3300	0	0	1600	0–3400
H2a2a	7900	3300–12,600	6500	3600–9400	10,800	6900–14,800
H2a2a1	4500	3200–5900	4600	2400–6800	5000	3300–6700
H2a2a1d	2600	0–6400	0	0	2300	0–5200
H2a2a1b	2000	0–4200	2000	0–5900	2200	0–4800
H2a2a1a	400	500–8200	2600	0–7800	3300	800–5800
H2a2a2	5200	400–10,200	4000	0–8400	4900	0–17,300
H3p	9700	1700–18,000	5200	0–15,500	10,600	1800–19,800
H3p+146	1300	0–3800	0	0	1100	0–500
H4a1a-195C	10,000	3400–16,700	14,900	0–30,600	8000	4400–11,600
H4a1a3	5700	400–11,300	3100	0–9300	5400	0–11,100
H4a1a3a	3200	0–7100	3900	0–11,700	2800	0–10,500
H4a1a3a+513	0	0	0	0	0	0
H4a1a4	7900	1400–14,700	10,700	0–24,400	6300	3000–9700
H4a1a4a	2600	0–5600	0	0	3000	0–6700
H4a1a4b	3000	1600–4400	3300	700–5900	3100	1500–4700
H5	12,000	8000–16,100	12,800	6300–19,300	12,500	5500–19,800
H5-73G	7000	1000–13,200	5200	0–12,500	8900	1400–16,800
H5-73G-16167-16274	1300	0–3800	0	0	1600	0–4700
H5a7	5200	1200–9300	5200	0–11,500	4600	0–9700
H5c	7600	3300–12,000	96000	1100–18,200	8200	3100–13,200
H5c1	5200	400–10,200	7900	0–19,400	5600	600–10,700
H5c2	500	0–11,800	0	0	2000	0–5600
H5c2-16304C!	1300	0–3800	0	0	800	0–3300
H6a1a	6900	5300–8600	7100	4100–10,100	700	5100–8800
H6a1a1	8600	2600–14,800	15,700	1300–30,200	5700	2300–9200

**H6a1a1a**	1300	0–3800	3900	0–11,700	800	0–2500
**H6a1a3**	2600	300–4900	1600	0–4700	3100	0–6300
**H6a1a5**	2600	0–5300	1600	0–4700	3100	0–6300
**H7**	9700	7300–12,100	11,700	6800–16,600	12,900	7200–18,800
**H7+152**	4700	1600–7800	6300	100–12,500	6000	2000–10,200
**H7c**	8100	4600–11,600	7300	2500–12,200	10,400	6000–15,000
**H7c1**	4800	700–9000	4000	0–8400	5300	0–11,000
**H7c2**	900	0–2600	0	0	1200	0–10,300
**H7e**	3100	600–5600	4700	0–10,100	3800	700–7000
**H11**	13,700	6300–21,300	9400	3900–14,800	11,600	5900–17,400
**H11a**	8500	5400–11,700	8400	2800–14,100	6900	4900–8900
**H11a1**	3700	300–7200	2200	0–5400	3500	300–6800
**H11a2**	11,400	4500–18,600	6600	2400–10,700	6300	4200–8500
**H11a2a**	6700	3100–10,300	6700	1500–11,800	5000	2700–700
**H11a2a1**	3100	0–6700	3100	0–7500	2700	0–5800
**H11a2a2**	4300	500–8200	300	0–7800	2600	100–5000
**H11b1**	6100	2300–10,000	3900	0–8400	6500	1100–12,000
**H11b1-16261T**	1300	0–3800	0	0	1400	0–4100
**H13a1a1**	7200	4700–9700	4200	1700–6800	7200	4800–9600
**H13a1a1+150**	3500	100–6900	5200	0–12,500	4700	40–9100
**H13a1a1d**	4200	0–8800	6300	0–18,600	2800	100–5600
**H13a1a1a**	6800	2400–11,300	3900	500–7400	6200	3400–9000
**H13a1a1e**	3500	100–6900	2600	0–7800	4000	300–7900
**H13a1a1b**	3900	0–8400	0	0	4300	0–8800
**H15**	13,400	7200–19,800	22,200	5600–38,800	14,400	9400–19,500
**H15a**	10,400	4500–16,600	15,300	0–30,800	11,400	7100–15,900
**H15a1**	7900	4500–11,400	7900	2000–13,800	9000	6700–11,300
**H15a1a**	5200	400–10,200	0	0	5600	900–10,500
**H15b**	11,100	6300–16,000	11,800	3300–20,00	12,000	7200–16,800
**H26**	10,600	5300–16,000	19,700	5800–33,500	10,500	5500–15,600
**H26a1**	6400	2900–10,100	7900	2400–13,300	6000	2000–10,000
**H26b**	12,000	4100–20,300	23,600	4700–42,500	7100	2800–11,100
**H26c**	3200	0–7100	2000	0–5800	3900	0–16,800
**H26c+16129**	0	0	0	0	0	0
**H40**	5600	2400–8900	5600	0–11,500	7500	400–14,900
**H40b**	1300	0–3800	0	0	1400	0–12,800

Supplementary Table S9. The source of Ashkenazi mtDNA lineages based on 836 control-region sequences[1,2,11,45]. Each sequence was cross-referenced with the mitogenome phylogeny[36], and either assigned to a probable European, Near Eastern or Asian source or left unassigned due to ambiguity in the phylogeographic distribution. Haplogroup H assignments based on whole mitogenomes (see Supplementary text). "HV" includes HV1, a single HV2 lineage, two HV13 lineages and five putative HV5 lineages; "U" includes U1, U2, U3, U4, U6 and a single U8b1 lineage; "others" include single N1c, N9a and two putative K1a4 lineages.

Source	H	HV	HV0	I	J	K1a1b1a	K1a9	K2a	L2a1l	M1a1b	N1b2	R0a1a	T	U	U5	U7	W	X	M/A	Others	Input
**Europe**	20.5	—	4.1	1.3	6.3	20.2	6.3	5.3	—	0.7	9.2	—	3.0	0.2	2.0	—	1.6	—	—	—	80.7
**Near East**	0.8	3.6	—	—	0.1	—	—	—	—	—	—	2.0	0.2	—	—	1.1	—	—	—	0.4	8.3
**Asia**	—	—	—	—	—	—	—	—	—	—	—	—	—	—	—	—	—	—	1.0	0.1	1.1
**Unassigned**	1.6	1.4	—	—	0.4	—	—	—	1.4	—	—	—	1.6	2.8	—	—	—	0.8	—	—	9.9
**Total**	22.9	5.0	4.1	1.3	6.8	20.2	6.3	5.3	1.4	0.7	9.2	2.0	4.8	3.0	2.0	1.1	1.6	0.8	1.0	0.5	100.0

Supplementary Note 1

Phylogeography of haplogroup U8: context of the Ashkenazi haplogroup K founders

Here we present the context for the Ashkenazi haplogroup K lineages, providing the detailed phylogeographic evidence supporting a deep ancestry for the Ashkenazi founder clades within Europe.

Haplogroup K is by far the largest subclade within haplogroup U8. The phylogenetic tree (Supplementary Data 1, with its main features summarised schematically in Fig. 1) shows the complete U8 phylogeny, comprising 956 whole mitogenomes, including 909 from haplogroup K (Supplementary Data 3). The tips are colour-coded for geographical information and age estimations are provided at the main nodes (details in Supplementary Data 2). There are four estimates for each node: based on ρ with mtDNA synonymous sites only, ρ and ML (using PAML) with whole mitogenomes, with a correction for purifying selection[38], and Bayesian estimates using BEAST[46], based on several internal calibrations within haplogroup U[40].

The estimates are broadly similar (allowing for the greater imprecision of the synonymous estimates), although those from BEAST tend to be rather lower. It is worth noting that the Bayesian estimates are completely independent from those using ρ and ML, using a wholly different approach to both calibration and estimation. However, they were necessarily carried out with only a subset of the data (398 sequences) due to the limitations of the software, and given the necessary assumptions of the Bayesian approach (and, conversely the lack of any mutation model for the ρ estimates) we regard the ML values as the most reliable.

The coalescence time of U8 is ~52 ka. Its age and distribution (Supplementary Fig. S1; Supplementary Table S1) suggest that U8 is one of the few extant mtDNAs dating to the time of arrival of anatomically modern humans in the Near East, ~50

ka[47,48], and may have been one of the earliest mtDNAs to arrive in Europe – a prediction strikingly supported by the isolation of a U8 lineage, most likely pre-U8b (as it includes one of the three variants diagnostic of U8b), alongside probable pre-U5 lineages (with two HVS-I variants characteristic of U5), from human remains at Dolní Věstonice in the Czech Republic dating to ~31.5 ka[49]. The deepest split separates U8a, which dates to ~22 ka, from U8b, dating to ~47 ka. U8a is very rare, and appears to be exclusively European. U8b1, dating to ~44 ka and identifiable in the HVS-I database, is scattered at very low frequencies across west Eurasia, with a focus around the eastern Mediterranean and Southwest Asia. Its sister clade within U8b, the much more frequent haplogroup K, is ubiquitous throughout western Eurasia.

The HVS-I data show that haplogroup K as a whole has frequency peaks in north-west Europe and between south-east Europe, Anatolia and the Levant, dipping in central and north-central Europe (Supplementary Fig. S1; Supplementary Table S1). The diversity distribution is fairly similar; there is a minor peak in south-west Europe with the major peak in south-east Europe/south-west Asia (Supplementary Fig. S2; Supplementary Table S2). However, the main subclades of haplogroup K are not visible in HVS–I data, so further investigation of their distribution using the HVS–I database alone is not possible.

Using the whole-mitogenome evidence, we can see that haplogroup K dates to ~36 ka and splits into two primary subclades, K1 and K2 – although a single sequence from the South Caucasus appears to fall into a third basal branch[50]. This might hint at a Near Eastern origin for haplogroup K as a whole[51]. A Near Eastern origin for haplogroup K might also be suggested by both the Southwest Asian focus of its sister clade U8b1 and the HVS–I diversity pattern (Supplementary Fig. S2). Given the timing of the appearance of haplogroup K, just prior to the global climatic downturn, an origin

in the Near East – which acted as a major reservoir for mtDNA variation during the glacial period[30,52] – might also more plausibly account for its survival than an origin in Europe.

None of these arguments is compelling, however, and on the other hand, the majority of the major subclades within haplogroup K appear to have arisen within Europe (see below), with K1a the only likely exception, which points to a European origin for haplogroup K (Fig. 1). Furthermore, although the present distribution of U8b1 is widespread and uninformative, the probable pre-U8b lineage identified at Dolní Věstonice shows that U8b (of which haplogroup K is a subclade) has a presence in Europe dating back more than 30 ka[49]. Overall, we regard the place of birth of haplogroup K as unresolved, although Europe seems more likely than the Near East, given the rather weak evidence presently available. On the same grounds, the same is true for K1.

Haplogroups K1 and K2 both date to ~29 ka, the cooling period from ~33ka prior to the LGM (from ~26.5 ka to ~19.5 ka[53]). Both divide into three further basal subclades, in each case a major one (K1a and K2a) and two minor ones (K1b, K1c; K2b, K2c). By far the most frequent of these subclades is K1a, dating to the end of the Last Glacial Maximum (LGM) at ~20 ka, and comprising numerous subclades of various ages, some of which are found predominantly in the Near East and some in Europe. K1b and K2c both date to the pre-LGM downturn, ~27 ka; K1c and K2b both date to the Late Glacial, ~17–18 ka; and K2a dates to the Neolithic, ~8.5 ka.

All of the subclades, apart from K1a, are found mainly in Europe, with occasional lineages appearing in the Near East (K2c is extremely rare and remains enigmatic). All four of the minor subclades bifurcate again, with most multifurcating nodes appearing only within the last 10 ka, but within K1b some arise earlier, ~10–25

ka. Although these subclades appear to be mainly European, one nested subclade within K1b, K1b1c, dating to ~8 ka, is seen mainly in the Near East, suggesting dispersal from Europe to the Near East in the early Holocene.

Aside from this, there are several isolated Near Eastern lineages within predominantly European clusters, suggesting much more recent individual migration events from Europe into the Near East – what one might refer to as “erratics” or “accidentals”, by analogy with ornithology. These include a single lineage from Iraq within K2b1; a sequence shared between an Anatolian and an Indian and a related sequence in Pakistan within a subclade (K2a5) that otherwise includes four west Europeans (with the remainder of K2 including individuals only from Europe and the US, plus one Algerian Jew); and single instances from Anatolia and Armenia amongst 12 European and two US lineages in K1b2. There is also single (urban) Anatolian lineage within K1a4a1, which otherwise includes 52 European, one North African and seven US lineages. There are likewise several instances of European lineages nesting within Near Eastern subclades of K1a3 and K1a4, mostly involving exchange along the Mediterranean, some of which may be very recent (*e.g.* between Greece and Turkey) and some ancient[30]. On the other hand, within K1a, several Near Eastern lineages emerge directly from the root, as well as a single Armenian lineage at the root of K1a1, a contrasting nesting pattern consistent with a Near Eastern origin for K1a.

A small cluster, K1d’e’f, which is defined by a single transition at the fast-evolving control-region position 16362, includes several European lineages and the Tyrolean Iceman’s sequence[54,55], dating (by radiocarbon) to the Alpine Late Neolithic and recently labeled as K1f [36]. If these lineages are truly monophyletic, this would likely represent a further basal European subclade, dating to the onset of the last glacial period (close to the age of K1 and K2, at ~26 ka), which has become almost extinct in

the present day. Otherwise we would have here up to four more basal lineages, each of which currently includes only European samples.

The time depth and geographical distributions of K1b, K1c, K2a and K2b suggest that they all arose within Europe, between the LGM and the Neolithic. This in turn suggests that K2 itself may have arisen within Europe, early during the LGM. All the subclades (except the very minor K2c) show signs of multifurcation, suggesting demographic expansion, during the Holocene. Indeed, there are three major signals of population growth detected amongst European U8 lineages in the skyline plots by BEAST (Supplementary Fig. S3). The earliest occurs in the Late Glacial, between ~17 ka and 13 ka, indicating about four-fold expansion – much weaker than that for the huge burst of growth in the Near East in the same period (see below). On the other hand, there are two distinct episodes of Holocene growth seen in Europe that are not evident in the Near East. One occurs with the Neolithic, beginning ~9 ka, reflecting the burst of branching especially in K2, K1c, and K1a4a1 (the main European subclade of K1a4), and indicating a six–fold expansion between 9.3 and 4.5 ka. The second rather strikingly begins in the Bronze Age~3 ka, indicating a seven-fold expansion between 3.3 ka and five hundred years ago (Supplementary Fig. S3). This late Holocene growth reflects many multifurcations dating to roughly this age in K2a, K2b, K1c, K1b2 and K1a, including several within K1a4a1 and K1a4d, and it mirrors the pattern seen in haplogroup U5 in Europe[40].

Interestingly, a pattern of Neolithic growth from BEAST has recently been used to suggest an expansion of haplogroup H from the Near East in the Neolithic[56], pointing to the pitfalls of ignoring the phylogeographic signals in the data. A deep, pre-Neolithic European ancestry for the great majority of European mtDNAs is in fact consistent with

the high-resolution autosomal SNP (single nucleotide polymorphism) evidence, which implies a predominantly indigenous origin for most European populations[3].

Thus the Ashkenazi founder clade K2a2a1, dating to ~1.5 ka, nests within European lineages dating to at least the Neolithic (the age of K2a) and most likely back to before the glacial period, ~30 ka (the age of K2) (Supplementary Data 1; Fig. 4).

By far the most common and complex subclade of haplogroup U8 is K1a, with lineages distributed throughout Europe, the Near East and the Caucasus. K1a dates to the end of the LGM, ~20 ka, and radiates a large number of basal subclades at this point, suggesting an early Late Glacial expansion; moreover, most of the subclades themselves suggest Late Glacial expansions, ~13–18 ka. Out of 18 putative basal subclades identified here (counting those with >2 lineages present in each), 14 (including all of the most frequent) date to the Late Glacial, one to the immediate postglacial, and three to the Neolithic or post-Neolithic periods.

Several of the K1a subclades appear to have arisen within Europe and others in the Near East. K1a1 dates to ~18 ka and is essentially European in the whole-mitogenome tree (the only exceptions alongside 62 European, six US and 27 Ashkenazi sequences being solitary Armenian and Indian lineages), but control-region/typing data point to the presence of the K1a1* paragroup in the Near East as well as Europe, at similar frequencies (~0.5%)[2]. The early burst of multifurcation may therefore reflect a Late Glacial origin in the Near East. Its major subclade, K1a1b1 (Fig. 2), however, appears to be exclusively European/North African, with a geographical focus on the western Mediterranean, and dates to 11.7 ka. K1a1 therefore most likely dispersed to Europe during the Late Glacial, sometime between the age of the common ancestor, ~18 ka, and 12 ka. The much more recent Ashkenazi founder clade K1a1b1a therefore has a very deep European ancestry, in sharp contrast to HV1b2, for example (Fig. 5).

Along with K1a1, K1a4 is the largest K1a subclade. K1a4 is found across Europe and the Near East, but the phylogenetic nesting patterns suggest a Near Eastern source. Most of the deepest lineages in K1a4, which dates to ~18 ka, are also from the Near East, and the overall diversity (measured by ρ) is higher in the Near East than Europe (Supplementary Data 1–2; Supplementary Table S3). It accounts for the major signal of population expansion in the Near East detected in the skyline plots by BEAST across the Late Glacial period, around 50-fold from 20 ka to 10 ka, (Supplementary Fig. S3). K1a4b and K1a4c, both dating to the Late Glacial, along with many basal K1a4 lineages, are largely restricted to the Near East. K1a4a also includes several basal Near Eastern lineages, along with an Ashkenazi lineage that evidently heralds from the Near East – the only example from haplogroup K for which this is the case.

However, K1a4a encloses a large, mainly European subclade, K1a4a1, dating to ~11 ka, nesting within deeper Near Eastern and North Caucasian lineages dating to ~16 ka, suggesting (similarly to K1a1b) a dispersal into Europe during the Late Glacial or immediate postglacial. K1a4a1 shows signs of an early Holocene starburst expansion in Europe, alongside a number of smaller subclades such as K1a4e and K1a4f1. Further nested subclades within K1a4a1 (K1a4a1a, b and c) date more squarely to the European Neolithic, ~8 ka, but must have arisen within Europe after the earlier arrival from the Near East. K1a4d radiates much more recently in central and north-west Europe, ~3 ka.

K1a3 shows a similar pattern. Like K1a4, the basal lineages suggest that K1a3 also arose in the Near East in the Late Glacial, ~16 ka, with a subclade, K1a3a, dispersing into Europe by ~14 ka. There is a rare, single Ashkenazi Jewish lineage, and also a Moroccan Jewish lineage, within this European subclade. These were evidently assimilated within Europe separately from the main three haplogroup K founders (and

from the single K1a4a lineage mentioned above), and evidently also separately from each other, since they fall into different subclades of K1a3a.

This pattern in K1a3 (and probably also K1a4a) appears analogous to recent findings for a large fraction of haplogroup J and T lineages[30]. Like those, it highlights the Near East as an important glacial refugium for European as well as Near Eastern hunter–gatherer populations, with dispersal into Europe at the end of the glaciation and then expansion within Europe in the early Holocene and with the onset of the Neolithic.

K1a2, dating to ~17 ka, may have a similar history again. It is predominantly European but with a single Armenian lineage at its root, possibly indicating a Near Eastern source and an early Late Glacial dispersal into Europe: K1a2a and K1a2b are both wholly European and date to the Neolithic and Late Glacial respectively. There is also a derived Anatolian lineage within the minor K1a2d, suggesting back-migration from Europe.

Most of the other basal K1a subclades are predominantly or wholly Near Eastern, with mainly Late Glacial coalescence times, with in some cases derived, less ancient nested European subclades – strongly implying a Near Eastern origin for K1a as a whole. Mainly Near Eastern K1a subclades include K1a5, K1a6, K1a8, K1a12, K1a17, K1a18, K1a19, K1a28 and K1a29; and there are also a number paraphyletic K1a* lineages found in the Near East, again suggesting that K1a most likely arose in the Near East (although they are also quite frequently found in southern Europe).

Other than K1a1a and K1a1b1, the main European deep-rooting putative K1a subclades are K1a13’16’31 (defined by the fast site 195) and K1a9’10’15’26’30 (defined by the fast sites 195 and 16093). Several ambiguities in fact remain in the K1a phylogeny, relating to the use of these positions in the reconstruction. In particular, the rate at site 16093 appears to be elevated, similar to the elevation at particular sites in

other haplogroups such as T[57]. The possible K1a13’16’31subclade, unified in our tree by a transition at position 195 in HVS–II, would have a north-Mediterranean focus and date to ~11 ka. The second putative European subclade, unified by both a transition at position 195 in HVS–II and a transition at 16093 in HVS-I (K1a9’10’15’26’30), which includes the major Ashkenazi founder clade, K1a9, might equally parsimoniously derive either in the way we have presented the tree (Supplementary Data 1; Fig. 1; Fig. 3) or alternatively via K1a13’16’31, defined by the transition at site 195 (Supplementary Fig. S4). Either alternative would imply an early Holocene, west European (perhaps Mediterranean) ancestry. In the former case, the last split with Near Eastern lineages (K1a28 and K1a20) is ~17 ka, and the dispersal into Europe would be sometime between 17 ka and 11 ka, the age of K1a9’10’15’26’30. In the latter case, the putative subclade is basal, and the dispersal would be sometime between the age of K1a13’16’31 (found only in Europe) and K1a itself (originating in the Near East).

Given that both positions 195 and 16093 are in top ten fastest positions in the mtDNA[38], it is also theoretically possible that K1a9 might (less parsimoniously) represent a distinct branch within K1a, with additional “hidden” recurrent mutations at sites 195 and 16093. If this were the case, a Near Eastern ancestry for K1a9 would remain a (remote) possibility. However, it is noticeable that the two clusters defined respectively by variants at both positions 195 and 16093, and 195 alone, respectively, lack any Near Eastern lineages within them. Especially given the prevalence of paraphyletic K1a lineages in the Near East, it seems extremely unlikely that none of them would have given rise to any Near Eastern derivatives, if these putative subclades were not monophyletic.

A Near Eastern ancestry was in fact proposed by Behar et al.[2] in order to explain K1a9 haplotype sharing in one Iraqi Jew and one Syrian Jew. Given the clustering

ambiguity, we cannot entirely rule this out, but Syrian Jews have Sephardic ancestry, and it would be unwise to draw conclusions from a single Iraqi sample, which may be the result of an "accidental", quite possibly recent, instance of gene flow.

In conclusion, the phylogeographic patterns strongly imply that of the three major Ashkenazi founders, K1a1b1a most likely traces its ancestry within Europe to the early Holocene, and K2a2a1 to at least the mid-Holocene and most likely much earlier, back to the glacial period ~30 ka. For the reasons mentioned above, K1a9 is less definite, but it most likely derives from K1a9'10'15'26'30, which would trace to Europe in the early Holocene, at a similar time and place to K1a1b1.

A case might be made for a Levantine origin for the haplogroup K lineages on grounds of an insufficient Near Eastern sample database, so that the Ashkenazi lineages might simply have not yet been sampled in the Near East: a reservoir of haplogroup K variation, such as that identified by Shlush et al.[52] in the Druze, might still be awaiting discovery. However, the distribution of Near Eastern lineages across the haplogroup K tree suggests that this is very unlikely. Near Eastern lineages, although diverse, are nevertheless not scattered throughout the tree, but fall mainly within particular subclades of haplogroup K1a, and are strongly concentrated in these particular clusters. The main exception, K1b1c, may be the exception that proves the rule, since it looks like a clear case of a Holocene migration from Europe.

Given this pattern, it is difficult to imagine that there could be hidden Near Eastern reservoirs carrying three major clusters identified to date only in Europe, given also that 95 mitogenomes from the Near East/South Caucasus are included and that there is no suggestion of significant further variation in the much more extensive control-region dataset. Potential reservoirs with high haplogroup K frequencies (such as Georgia) follow the Near Eastern pattern and show no sign in the mitogenome data of

harbouring potential Ashkenazi founders. Chuvashia is another case in point (see Supplementary Note 2). Out of the 107 Near Eastern/South Caucasian lineages, there are only a small number of cases, detailed above, that "invade" the predominantly European subclades, and six of these are from Armenia or Anatolia, areas well known from previous mtDNA studies, as well as the historical record, to have experienced heavy influxes from Europe in the past few thousand years[23]. By comparison, for the Ashkenazi founder clades, K1a1b1 includes 37 European lineages, K1a9, 34 Europeans and one North African, and K2a2, eight Europeans; none includes a single Near Eastern lineage.

Moreover, mtDNAs from Samaritans, who can reasonably be expected to have shared an ancestral Hebrew gene pool with Palestinian Jews and who, consistent with this, cluster with modern Druze and Lebanese in autosomal analyses[3], lack any haplogroup K lineages, or indeed the other more common Ashkenazi founder mtDNAs (in N1b, H or J), whilst carrying Near Eastern lineages (predominantly T2h, U7 and H20)[58,59] that include several that are in fact closely related to minor Ashkenazi lineages of putative Near Eastern origin (U6a, R0a)[30,31].

The question arises as to why an assimilation founder event might draw in several lineages from a single haplogroup (K) from a presumably diverse source population in Europe. However, we would caution against assuming that the source must have been enriched for haplogroup K. We have now shown that N1b2 underwent a similar process (main text; Fig. 6; Table S5), and numerous minor lineages may have been assimilated at the same time (with some lost later by drift). Moreover, whereas K1a1b1a and K1a9 were most likely assimilated in Mediterranean/Western Europe, the third major K founder, K2a2a1, with its slightly younger age estimate and its nesting within a German lineage, may have been assimilated in central Europe slightly later, again along with

numerous minor lineages (such as many of those from haplogroup H; see below). The survival of three distinct haplogroup K founder lineages through the early bottleneck may simply have been due to chance.

Supplementary Note 2

Southern Russian mtDNA lineages and the "Khazar hypothesis"

There is no evidence in the mtDNA pool to support the contention that lineages might have been recruited on a large scale from the North Caucasus and/or Chuvashia regions of southern Russia, as would be predicted by the "Khazar hypothesis"[16]. Not only are the subclades of haplogroup K that gave rise to the three major Ashkenazi founders absent, and N1b virtually so, but there is little evidence for any assimilation of the more minor Ashkenazi lineages either, most of which comprise mtDNAs found primarily in Europe. Extant southern Russians from these regions carry either distinct subclades of haplogroups from those found in the Ashkenazim (for example, amongst the more common west Eurasian clades such as haplogroups H, J and T lineages) or they lack the other haplogroups found in the Ashkenazim altogether.

There are 208 sequences from the North Caucasus in the HVS-I database (excluding Kalmyks who arrived from China in the seventeenth century AD), of which eight belong to haplogroup K[23]. We fully sequenced seven of these and partially sequenced the eighth, in order to resolve their haplogroup status. All belonged to Near Eastern subclades, and none belonged to one of the three Ashkenazi founder clades. Two belonged to K1a3, two to K1a12a, and the remaining four belonged to K1a4 (including two belonging to K1a4a3 and one to K1a4g). There is a single N1b1 sequence, but no case of N1b2.

~25% belong to haplogroup H but, unlike those in the Ashkenazim, most do not fall within typically European H subclades, with only a few H5 and H6 and a single H1b. HV1a is present, but not HV1b, which is the main HV1 subclade in the Ashkenazim. There are several examples of haplogroups C1 and D, as well as G2a and some Asian haplogroup N lineages, none of which is seen in the Ashkenazim. Similarly, most of the haplogroup J lineages in the North Caucasus are distinct from those in Europe, with far fewer J1c, which is the main European J subclade. There is, however, one exact match to the J1c7a1 sequence, also found in Eastern Europe, which may therefore be a potential candidate for a "Khazar" lineage. However, this lineage is found more commonly in west and central Europe, and the nesting pattern suggests that those in Eastern Europe may have spread from the Ashkenazim community into their neighbours, so the possibility remains remote. Again, haplogroup T in the North Caucasus lineages are markedly different, with the exception of the ubiquitous T1a, with the Ashkenazi/European T2b and T2a1b forming a very small minority of North Caucasus T2 lineages.

The haplogroup U3 mtDNAs in the Caucasus are mostly U3b3, whereas those in the Ashkenazim are U3a. There is no U5b in the North Caucasus and the U5a lineages are not similar to those in the Ashkenazim. There are no haplogroup U7 or U6 lineages and those belonging to haplogroups I, W and X are also distinct. There is no haplogroup V7a, only V10a. M1a1 is present, but no M1a1b, and the situation is similar for U1b1. Overall, there is very little case to be made for any assimilation into Ashkenazi communities from this region.

The language of the Chuvash, who now reside slightly to the northeast in the Middle Volga region, is thought to be close to that spoken by the Khazars, and the

region is considered to have been a sink for dispersals after the fall of the Khazar empire[60]. In our small sample of 36 Chuvashian mtDNAs, six (16.7%) belonged to haplogroup K. However, the four that we were able to test belonged to K1a4a1h, K1a5a and pre-K2a8, and a fifth can be identified as K1c1e. There are no N1b lineages, and few other lineages that might suggest any potential contribution to the Ashklenazi mtDNA pool: the only HVS-I matches are with the geographically extremely widespread root types of H5, H6a1a and V (3% of Ashkenazi lineages in total). Thus, despite the high frequency of haplogroup K, this region too cannot be considered a potential important source for the Ashkenazi maternal lineages.

Supplementary Note 3

Minor Ashkenazi lineages: haplogroup H

There are 29 Ashkenazi haplogroup H whole mitogenomes currently available (Supplementary Table S7). This compares favourably with the 40 from haplogroup K (overall 32% in the Ashkenazim, compared with haplogroup H at 23%), although the haplogroup H lineages are considerably more diverse. The present whole mitogenome Ashkenazi database might potentially be biased, but cross-checking of these lineages with the much larger Ashkenazi control-region database (where, for haplogroup H, n = 191) confirms their European distribution and suggests that the available mitogenome data from Ashkenazim give a fair picture of the variation.

The control-region distributions for all Ashkenazi lineages (n = 836) are summarised in Supplementary Table S6. The overall frequency of haplogroup H in the Ashkenazi database is 23%, but this varies from only ~15% in Western Europe to >30% in parts of Eastern Europe. For comparison, haplogroup H is found in present-day

Europe at a rate of ~40%, the North Caucasus at 27%, and the Near East at 19%. Its major subclade, H1, varies from ~20–25% in Iberia to 10–15% in central and Eastern Europe to <10% in the North Caucasus and <5% in the South Caucasus/Near East, and H3 is almost entirely restricted to Western Europe[23,61-64].

The overall distribution of Ashkenazi haplogroup H control-region lineages is thus broadly similar to those of Europe and the Near East. Nevertheless, Ashkenazi haplogroup H lineages are highly distinctive in important respects. Virtually all of the Ashkenazi haplogroup H control-region sequences are found in the extant European mtDNA pool, but most are elevated to higher frequencies in the Ashkenazim. The fraction of singletons is correspondingly lower (17% *versus* 49% in a sample from Germany), as is the fraction of unique haplotypes overall (17% *versus* 37%).

Haplogroup H, like haplogroup K, carries only a weak phylogenetic signal in the control region (especially HVS-I), which means that we cannot pinpoint the position of all of the Ashkenazi haplogroup H lineages in the control region database in the way that we can for many of the lineages in other haplogroups (see below). We have therefore estimated the fraction of Ashkenazi lineages within haplogroup H that can be allocated to a European source using the 29 mitogenomes. Even so, at least a third of the control-region lineages can be assigned to H1 and H3, and another 12% to the predominantly European H5. More specifically, almost a quarter can be allocated to specific subclades within haplogroup H1 in the global mitogenome tree (H1b, H1e4a, H1aj1) (Fig. 7) with another quarter allocated to H2e2a1, H3*, H5*, H5c2, H6a1a*, H6a1a1a, H10a1b, H11 and H15b (see Fig. 8; Supplementary Figs. S5-S10). Of these, only H5–73G (Supplementary Fig. S5), H6a1a1a (Fig. 8) and H15b (Supplementary Fig. S9) can plausibly be ascribed a potential Near Eastern origin.

There are several subclades within H5 (Supplementary Fig. S5), which again nest with west European lineages; and in H7 (Supplementary Fig. S6), which also appears to have arisen in Europe (although several distinct subclades have spread subsequently to the Near East). The latter (as for H1as and H40b: Supplementary Table S7) lack a clear control-region motif and are candidates for Ashkenazi control-region lineages lacking a distinctive motif.

H6 is predominantly European but is also seen in the Near East. A Slovakian Ashkenazi mitogenome falls alongside an Iranian Jew into one subclade dating to the early Holocene, H6a1a1 (Fig. 8), which includes individuals from Arabia and the North Caucasus (and matching a Czech individual in the HVS-I database). This is seen in the HVS-I Ashkenazi database at 0.6%, widely dispersed from west to east European groups, and this intriguing pattern clearly calls for further study, but H6a1a has been found in a late Neolithic (Corded Ware) sample from central Europe (Fig. 8; see below)[29].

Further minor lineages belong to H11, H13a1a1, H15b, H26c and H40b (Supplementary Figs. S7–S10). Of these, only H15b (Supplementary Fig. S9) can be assigned a potential Near Eastern origin; H40b remains ambiguous, whilst all of the remainder nest deep within European lineages in the mitogenome tree.

There is also an mtDNA from a single Spanish Sephardic Jew available, belonging to H56 which, like the Ashkenazi lineages, also nests within west European lineages, and a Bulgarian Jew belonging to H25, for which it is difficult to locate a source as the subclade emerges directly from the root of haplogroup H, with no nesting discernible (Supplementary Table S7). There is, furthermore, an mtDNA from a Turkish Jew belonging to H1p, emerging directly from the root of H1, which again most likely derives from a European source (Supplementary Table S7). There is therefore a

tentative suggestion of a European source for many Sephardic haplogroup H lineages as well. Although there are few whole-mitogenome data for Sephardim, the suggestion of a common European source with Ashkenazim is consistent with the autosomal evidence[3,4,27].

Finally, we note the presence in the whole-mitogenome database of a single haplogroup H13a2 lineage from a B'nei Israel individual from Mumbai[2] (Supplementary Table S7). Despite the few Indian lineages on the haplogroup H tree, this mtDNA clusters into H13a2a with one from Andhra Pradesh[36,65]. H13a2 is otherwise a mixed European/Near Eastern clade that is difficult to recognise in the control region database, but the pinpointing of the Indian ancestry in this instance provides further corroboration for the phylogeographic approach adopted here.

There are 22 Ashkenazi founders in total within the current haplogroup H mitogenome tree (Supplementary Tables S7, S8). Since the founder subclades at present comprise very few sequences, they can necessarily only be dated very imprecisely, but even so half of them date to <2ka (Supplementary Table S8). The remainder all include non-Ashkenazim individuals that inflate the age in at least some cases (*i.e.* in cases where the founder event was within the subclade, rather than cases where the whole subclade was involved in the founder event, followed by gene flow into neighbouring communities). In particular, the Near Eastern subclade H15b dates to the early Holocene, but includes a basal singleton Ashkenazi lineage, which may perhaps represent an individual recent migrant from the Near East (see below).

Powerful independent evidence corroborating a deep European ancestry for these lineages comes from the study of prehistoric remains, recently achieved for a sample of 39 haplogroup H lineages from prehistoric central Europe[29]. Haplogroup H1, and in particular H1e, along with haplogroups H5 and H26, have been found in early

Neolithic remains, H3 and H7 in mid-Neolithic remains, H6 and H13 in the late Neolithic and H2, H4 and H11 in the early Bronze Age. Thus almost all of the clusters to which the Ashkenazi haplogroup H lineages belong are known to have been present in Europe by 3500 years ago – including some (such as H6) now present at appreciable levels in the Near East – confirming the phylogeographic predictions made on the basis of their extant distribution and molecular-clock estimates. The only lineages not present in the prehistoric sample (from the mitogenome tree evidence) are H15b, an early Holocene clade that may indeed have Near Eastern ancestry (but which can be identified from its HVS-II motif and is vanishingly rare in the control-region database) and H40b, which nests within mainly European lineages in the mitogenome tree.

If we conservatively regard H6a1a1 as potentially having a Near Eastern source, we can therefore assign 26 out of the 29 (89.7%) Ashkenazi haplogroup H mitogenomes to a European source. Given the phylogenetic ambiguity of control-region data for haplogroup H, as mentioned above, this is the best estimate we can provide at present. 89.7% of the 22.9% of the control-region Ashkenazi database comprising haplogroup H suggests an overall European source component of 20.5%.

Other minor Ashkenazi mtDNA lineages

There are numerous other minor founders in the Ashkenazi mtDNA pool, as can be seen in the large control-region database (summarised in Supplementary Table S6), relatively few of which have yet been subjected to whole-mitogenome sequencing on the scale of haplogroup H, let alone haplogroups K and N1b. On the other hand, there is far less ambiguity in the phylogenetic position of most control-region lineages than for haplogroups H and K, so most of the remaining lineages can now be pinpointed fairly closely on the mitogenome tree of extant lineages from Europe and the Near East.

Moreover, by combining the control-region and whole-mitogenome database for west Eurasians, most of those can be further assigned to a geographical source with some confidence (Supplementary Table S9).

After the four major founders and haplogroup H, **haplogroup J** is the next most common haplogroup in the Ashkenazim, at ~7% overall. As with the haplogroup H lineages, these provide further substantial corroboration for the importance of Europe as the primary source for Ashkenazi maternal lineages. Ashkenazi J lineages are unevenly distributed but are, like those of H, rarer in the west Ashkenazim (~5%) and more frequent in eastern groups, peaking in Russian/Belarusian Jews at ~16% (Supplementary Table S6). Again, a striking feature is elevated frequencies of rare haplotypes. (This reflects in reverse the distribution in the Ashkenazim of the main putative west Mediterranean lineages, K1a1b1a, K1a9, N1b2 and M1a1b.) The mitogenome tree for haplogroup J is well-known, with several Ashkenazi lineages included, and it is straightforward to locate most of the control-region sequences within it, as described in the main text[30]. As also discussed in the main text, at least 93% of Ashkenazi haplogroup J lineages, or 6.3% of total Ashkenazi lineages, have a likely European source (based on the frequencies in the control-region database, with lineages classified by cross-referencing to the whole-mitogenome tree[30]). Those within haplogroup J1c are shown in Fig. 10.

Haplogroup T lineages comprise ~4.8% of the Ashkenazim control-region database and of these at least 60% have a likely European source, thus the European component overall is at least 3%.

The **haplogroup M1a1** lineages all belong to a single subclade within M1a1b (based on the HVS-I variant at position 16289). M1a1b is restricted to the north Mediterranean[32], except for two individuals, from Saudi Arabia and Georgia

respectively, who cluster together in a separate subclade[66]. Although the single M1a1b founder is uncommon, comprising only 0.7% of the total Ashkenazi mtDNA database, it reaches 6% in Ashkenazim from France, consistent with a role as minor northwest Mediterranean founder. The **haplogroup I** lineages (I* and I5a, ~0.5%) have a likely European Mediterranean origin; although I1c1 (~0.8%) may have originated in the Near East, it is also present across the northern Mediterranean, including in remains from Neolithic Spain[31,67]. **Haplogroup W3** (~1.6%) appears to have been diversifying in Europe since Late Glacial times[31].

The **haplogroup U6** lineages (<1%) also have a likely Mediterranean origin, but whether it be the north, south or east coast is not clear. The HVS-I sequence is shared with Samaritans[59], suggesting a possible Levantine origin.

Haplogroup HV1b2 (~3% and mainly in the east, dating to 2.1 ka; with related lineages in Georgian and Iraqi Jews) clearly nests within an Armenian/Anatolian subcluster of the Near Eastern clade HV1b (Fig. 5; Supplementary Table S4). However, the control-region database shows that the clade and its nesting clade are also found in Mediterranean Europe, so although we assume here a Near Eastern source, strictly speaking this remains uncertain. There are several (0.2%) **haplogroup HV13** lineages also with a probable Near Eastern origin, with the remaining HV lineages in the Ashkenazim (1.1%) most likely (but not securely) belonging to **haplogroup HV5** – for which an origin cannot be estimated at present.

Haplogroup U7a5 is found across the range at very low levels (overall 1.3%) and has a likely origin in the Near East or South Caucasus. There is a single matching Iranian Jewish lineage in the database. **Haplogroup U1** (0.8%) comprises a number of diverse lineages of likely Near Eastern origin. The main **haplogroup R0a** lineage (~2%) is evidently basal within R0a1a and looks typically Near Eastern (although the

only exact match is in fact with a European). Jews have been mobile throughout their history, and some of these minor Near Eastern lineages may represent immigration of a few individuals within the last millennium from the Near East into Ashkenazi communities, rather than pointing to a common Near Eastern or Mediterranean source.

Supplementary References

45 Brandstätter, A. *et al.* Migration rates and genetic structure of two Hungarian ethnic groups in Transylvania, Romania. *Ann. Hum. Genet.* **71**, 791-803 (2007).

46 Drummond, A. J. & Rambaut, A. BEAST: Bayesian evolutionary analysis by sampling trees. *BMC Evol. Biol.* **7**, 214 (2007).

47 Higham, T. *et al.* Testing models for the beginnings of the Aurignacian and the advent of figurative art and music: The radiocarbon chronology of Geißenklösterle. *J. Hum. Evol.* **62**, 664-676 (2012).

48 Mellars, P. The earliest modern humans in Europe. *Nature* **479**, 483-485 (2011).

49 Fu, Q. *et al.* A revised timescale for human evolution based on ancient mitochondrial genomes. *Curr. Biol.* **23**, 553-559 (2013).

50 Schoenberg, A., Theunert, C., Li, M., Stoneking, M. & Nasidze, I. High-throughput sequencing of complete human mtDNA genomes from the Caucasus and West Asia, high diversity and demographic inferences. *Eur. J. Hum. Genet.* **19**, 988-994 (2011).

51 Irwin, J. A. *et al.* The mtDNA composition of Uzbekistan: a microcosm of Central Asian patterns. *Int. J. Legal Med.* **124**, 195-204 (2010).

52 Shlush, L. I. *et al.* The Druze: a population genetic refugium of the Near East. *PLoS One* **3**, e2105 (2008).

53 Clark, P. U. *et al.* The Last Glacial Maximum. *Science* **325**, 710-714 (2009).

54 Handt, O. *et al.* Molecular genetic analyses of the Tyrolean Ice Man. *Science* **264**, 1775-1778 (1994).

55 Ermini, L. *et al.* Complete mitochondrial genome sequence of the Tyrolean Iceman. *Curr. Biol.* **18**, 1687-1693 (2008).

56 Fu, Q., Rudan, P., Pääbo, S. & Krause, J. Complete mitochondrial genomes reveal Neolithic expansion into Europe. *PLoS One* **7**, e32473 (2012).

57 Malyarchuk, B. A. & Derenko, M. V. Molecular instability of the mitochondrial haplogroup T sequences at nucleotide positions 16292 and 16296. *Ann. Hum. Genet.* **63**, 489–497 (1999).

58 Shen, P. *et al.* Reconstruction of patrilineages and matrilineages of Samaritans and other Israeli populations from Y-chromosome and mitochondrial DNA sequence variation. *Hum. Mutat.* **24**, 248-260 (2004).

59 Bonné-Tamir, B. *et al.* Maternal and paternal lineages of the Samaritan isolate: mutation rates and time to most recent common male ancestor. *Ann. Hum. Genet.* **67**, 153-164 (2003).

60 Dalby, A. *Dictionary of Languages*. (Bloomsbury, 1998).

61 Ottoni, C. *et al.* Mitochondrial haplogroup H1 in north Africa: an early Holocene arrival from Iberia. *Plos One* **5**, e13378 (2010).

62 Achilli, A. *et al.* The molecular dissection of mtDNA haplogroup H confirms that the Franco-Cantabrian glacial refuge was a major source for the European gene pool. *Am. J. Hum. Genet.* **75**, 910–918 (2004).

63 Pereira, L. *et al.* High-resolution mtDNA evidence for the late-glacial resettlement of Europe from an Iberian refugium. *Genome Res.* **15**, 19-24 (2005).

64 Loogväli, E. L. *et al.* Disuniting uniformity: a pied cladistic canvas of mtDNA haplogroup H in Eurasia. *Mol. Biol. Evol.* **21**, 2012–2021 (2004).

65 Palanichamy, M. g. *et al.* Phylogeny of mtDNA macrohaplogroup N in India based on complete sequencing: implications for the peopling of South Asia. *Am. J. Hum. Genet.* **75**, 966-978 (2004).

66 González, A. M. *et al.* Mitochondrial lineage M1 traces an early human backflow to Africa. *BMC Genomics* **8**, 223 (2007).

67 Sampietro, M. L. *et al.* Palaeogenetic evidence supports a dual model of Neolithic spreading into Europe. *Proc. R. Soc. Lond., Ser. B: Biol. Sci.* **274**, 2161–2167 (2007).
